# Glioma-targeted oxaliplatin/ferritin clathrate reversing the immunosuppressive microenvironment through hijacking Fe^2+^ and boosting Fenton reaction

**DOI:** 10.1186/s12951-024-02376-w

**Published:** 2024-03-05

**Authors:** Xue Li, Ying Cheng, Zhifu Yang, Qifeng Ji, Menglei Huan, Weiliang Ye, Miao Liu, Bangle Zhang, Daozhou Liu, Siyuan Zhou

**Affiliations:** 1 Department of Pharmaceutics, School of Pharmacy, Air Force Medical University, Changle West Road 169, Xi’an, 710032 Shaanxi China; 2 Department of Pharmacy, Xijing Hospital, Air Force Medical University, Xi’an, 710032 China; 3 Key Laboratory of Gastrointestinal Pharmacology of Chinese Materia Medica of the State Administration of Traditional Chinese Medicine, Department of Pharmacology, School of Pharmacy, Air Force Medical University, Xi’an, 710032 China

**Keywords:** Glioma, Oxaliplatin, Ferritin, Ferroptosis, Immunosuppression

## Abstract

Glioma is easy to develop resistance to temozolomide (TMZ). TMZ-resistant glioma secretes interleukin-10 (IL-10) and transforming growth factor-β (TGF-β), recruiting regulatory T cell (T_reg_) and inhibiting the activity of T cells and natural killer cell (NK cell), subsequently forming an immunosuppressive microenvironment. Oxaliplatin (OXA) greatly inhibits the proliferation of TMZ-resistant glioma cells, but the ability of OXA to cross blood–brain barrier (BBB) is weak. Thus, the therapeutic effect of OXA on glioma is not satisfactory. Transferrin receptor 1 (TfR1) is highly expressed in brain capillary endothelial cells and TMZ-resistant glioma cells. In this study, OXA was loaded into ferritin (Fn) to prepare glioma-targeted oxaliplatin/ferritin clathrate OXA@Fn. OXA@Fn efficiently crossed BBB and was actively taken up by TMZ-resistant glioma cells via TfR1. Then, OXA increased the intracellular H_2_O_2_ level and induced the apoptosis of TMZ-resistant glioma cells. Meanwhile, Fn increased Fe^2+^ level in TMZ-resistant glioma cells. In addition, the expression of ferroportin 1 was significantly reduced, resulting in Fe^2+^ to be locked up inside the TMZ-resistant glioma cells. This subsequently enhanced the Fenton reaction and boosted the ferroptosis of TMZ-resistant glioma cells. Consequently, T cell mediated anti-tumor immune response was strongly induced, and the immunosuppressive microenvironment was significantly reversed in TMZ-resistant glioma tissue. Ultimately, the growth and invasion of TMZ-resistant glioma was inhibited by OXA@Fn. OXA@Fn shows great potential in the treatment of TMZ-resistant glioma and prospect in clinical transformation.

**Graphical Abstract:**

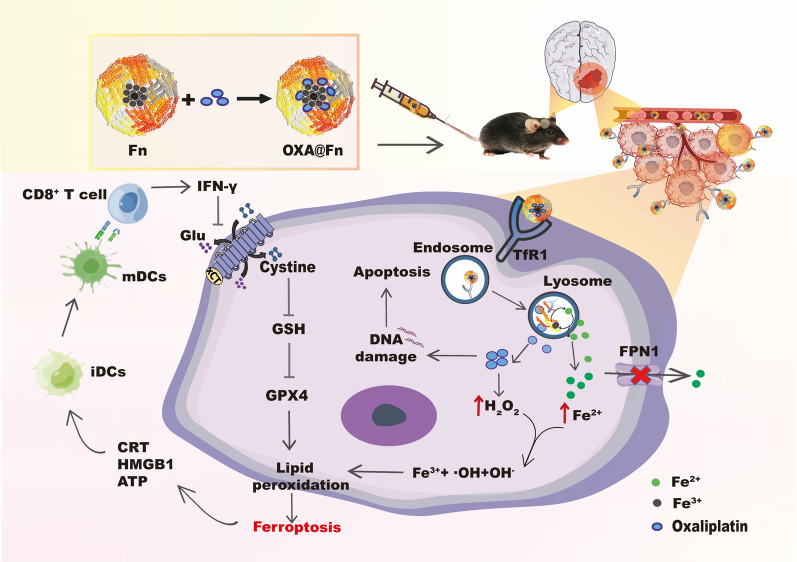

**Supplementary Information:**

The online version contains supplementary material available at 10.1186/s12951-024-02376-w.

## Introduction

Glioma is the most common primary brain cancer, accounting for 81% of malignant brain cancer. The annual incidence of glioma in China is approximately 7.01 per 100,000 for all populations [1]. At present, the standard clinical treatment for glioma is surgical resection combined with radiation therapy and temozolomide (TMZ) chemotherapy [2]. Because of invasive growth, it is difficult for glioma tissue to be completely removed by surgery. In addition, after 3 courses of TMZ chemotherapy, glioma develops resistance to TMZ, resulting in the recurrence of glioma. At present, the median survival of glioma patients is only 15 months [3]. We found that programmed cell death ligand-1 (PD-L1) in TMZ-resistant glioma tissue was high expressed, forming a significant immunosuppressive microenvironment [4]. TMZ-resistant glioma cells secrete interleukin-10 (IL-10) and transforming growth factor-β (TGF-β), thereby recruiting regulatory T cell (T_reg_ cell) and inhibiting the activity of cytotoxic T lymphocytes and natural killer cell (NK cell). T_reg_ cell inhibits the function of cytotoxic T lymphocyte by inhibiting the production of interleukin-2 (IL-2) and Interferon-γ (IFN-γ) and increasing the secretion of Th2 cytokines [5, 6]. In addition, TMZ-resistant glioma cells secrete immunomodulatory cytokines, which polarize M1 type glioma-associated macrophages (GAM) into an immunosuppressive M2 type GAM. M2 type GAM secretes multiple cytokines such as IL-10 to inhibit the function of cytotoxic T lymphocytes [7]. Therefore, by inhibiting the growth of glioma but improving the immunosuppressive microenvironment of TMZ-resistant glioma, the treatment of TMZ-resistant glioma is believed to be more effective.

Clinical study shows that most of recurrent glioma is resistant to TMZ [8]. Cisplatin, carboplatin and oxaliplatin (OXA) exhibit certain curative effect on recurrent glioma [9–11]. Cisplatin has significant ototoxicity and nephrotoxicity. Oxaliplatin does not cause nephrotoxicity or ototoxicity in clinical trials, and its myelosuppressive toxicity is less than that of carboplatin [12]. However, OXA shows weak ability to cross blood–brain barrier (BBB), so the concentration of OXA in glioma tissue is low, and the therapeutic effect of OXA on glioma is not satisfactory [13].

Ferritin (Fn), an endogenous protein consisted of 24 subunits, contains a hydrated iron oxide core and a cage structure of the protein shell [14]. In general, Fn plays an important role in maintaining iron homeostasis in the body [15]. Studies have shown that transferrin receptor 1 (TfR1) is highly expressed in cerebral capillary endothelial cells and TMZ-resistant glioma cells [16, 17]. Thus, Fn can efficiently cross BBB and target to glioma cells by binding with TfR1 [18]. In addition, drug can be easily loaded into Fn cavity through self-assembly of Fn subunit to increase the intracranial delivery of drug [19]. At present, Fn is considered as an excellent endogenous drug delivery vehicle, but few paper pay attention to the interaction between Fn and its loaded drug.

Ferroptosis is an iron-dependent programmed cell death [20]. Ferroptosis of cancer cell releases a variety of molecules such as high mobility group box 1 protein (HMGB1), calreticulin (CRT) and adenosine triphosphate (ATP) [21], which promote the maturation of dendritic cells (DC cell) in malignant cancer tissues [22]. The matured DC cells presents antigens of cancer cell to T lymphocytes, which subsequently activates effector T cell (T_eff_ cell) and finally promotes T cell mediated anti-tumor immune response and inhibits the growth of cancer in the body [23]. In addition, literature has shown when exogenous Fn is taken up by cancer cells, Fn is able to release Fe^2+^ in the lysosomes of cancer cell, thereby increasing the intracellular Fe^2+^ level [24, 25]. Therefore, Fn has potential in boosting Fenton reaction.

**Scheme 1:**
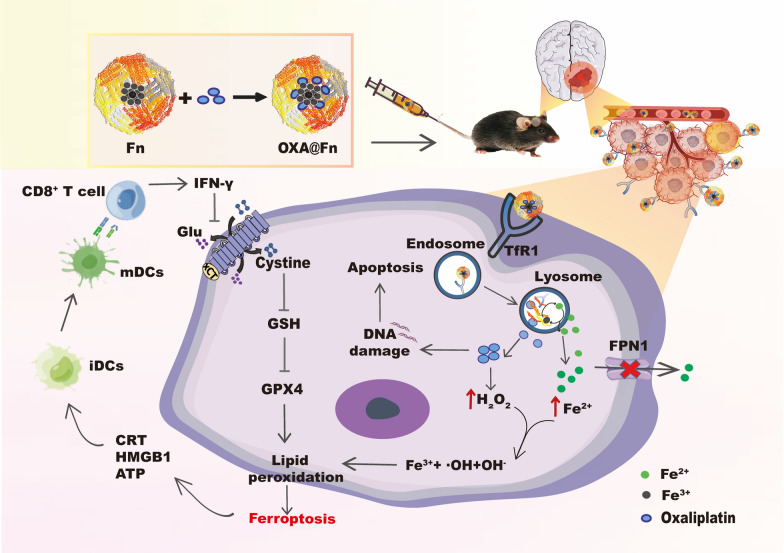
OXA@Fn crossed BBB and was specifically taken up by TMZ-resistant glioma cells through TfR1. H_2_O_2_ induced by OXA reacted with Fe^2+^ released by Fn to enhance the Fenton reaction, which subsequently boosted the apoptosis and ferroptosis of TMZ-resistant glioma cells and induced a strong T cell mediated anti-cancer immune response to inhibit the growth and invasion of TMZ-resistant glioma

Studies have shown that OXA can not only induce apoptosis but also promote the production of H_2_O_2_ in cancer cells [26–28]. In this study, OXA was loaded into Fn to prepare orthotopic glioma-targeted drug delivery system OXA@Fn. After OXA@Fn crossed BBB and was specifically taken up by TMZ-resistant glioma cells, H_2_O_2_ induced by OXA reacted with Fe^2+^ released by Fn to produce a large number of hydroxyl radicals, causing a bursting accumulation of lipid peroxides, which destroyed the redox balance in TMZ-resistant glioma cells and subsequently boosted the effect of ferroptosis of glioma cells. This induced a strong T cell mediated anticancer immune response in body. Consequently, the immunosuppressive microenvironment in orthotopic TMZ-resistant glioma tissues was reversed, and the growth and invasion of TMZ-resistant glioma were inhibited (Scheme 1).

## Materials and methods

### Materials

Oxaliplatin (OXA) was bought from Energy Chemical Technology co., Ltd (shanghai, China). Ferritin (Fn) was bought from Sigma-Aldrich Company (St. Louis, MO, USA). Glutathione peroxidase 4 (GPX4) antibody, cystine/glutamic acid reverse transporter (xCT) antibody, clusters of differentiation 44 (CD44) antibody, TGF-β antibody, E-Ca^2+^dependent cell adhesion molecules (E-cadherin) antibody, N-Ca^2+^dependent cell adhesion molecules (N-cadherin) antibody, matrix metalloproteinases-9 (MMP-9) antibody, clusters of differentiation 206 (CD206) antibody, ionized calcium binding adaptor molecule 1 (Iba-1) antibody, γ-H2AX antibody, Bcl-2 antibody and platelet endothelial cell adhesion molecule-1 (CD31) antibody were purchased from Abcam (London, Britain). Bax antibody, cleaved caspase-3 antibody was purchased from Cell Signaling Technology (MA, USA). TfR1 antibody, HMGB1 antibody and CRT antibody were purchased from Proteintech Co., LTD. (Wuhan, China). Antibodies used for flow cytometry were obtained from Biolegend (California, USA). Granulocyte–macrophage colony stimulating factor (GM-CSF) was purchased from Peprotech (Suzhou, China). Fe^2+^, glutathione (GSH) and H_2_O_2_ content assay kit were bought from Solarbio (Beijing, China). Tumor necrosis factor α (TNF-α), IFN-γ, IL-6, IL-12 and IL-10 enzyme linked immunosorbent assay (ELISA) kits were obtained from Shanghai Jianglai Biotechnology Co., Ltd (shanghai, China). 3-(4,5-dimethylthiazol-2-yl)-2,5-diphenyltetrazolium bromide (MTT) was obtained from Xi'an Kehao Biotechnology Co., Ltd (Xi’an, China). GL261 cells, BV2 cells and bEnd3 cells were purchased from CytoBiotech (Xi’an, China). TMZ-resistant GL261 cells (GL261/TR cells) were induced in our lab [3], and the drug resistance index of GL261/TR cells on TMZ was 22.74. C57 mice were provided by the Experimental Animal Center of Air Force Medical University (Xi’an, China).

### The preparation and characterization of OXA@Fn

OXA (2 mg) and Fn (6.4 mg) were dissolved in 2 ml distilled water. The above solution was stirred in a water bath at 60 °C for 4 h in dark room, and then it was cooled to room temperature. Finally, the solution was centrifugated for 10 min (3000×*g*) by using an ultrafiltration centrifuge tube with molecular weight cut-off of 50,000 Da to remove the unwrapped OXA.

The particle size, zeta potential and stability of OXA@Fn were determined by laser particle size analyzer (Malvern ZEN 3600 zeta particle size analyzer, Britain). A suitable amount of OXA@Fn was dropped onto the copper wire mesh, and the morphology and element mapping of OXA@Fn was observed by transmission electron microscopy (TEM, JEM-F200, Japan). X-ray photoelectron Spectrometer (XPS, Thermo Scientific K-Alpha, USA) was used to detect the surface elements of OXA@Fn.

The OXA content in OXA@Fn was determined by ultra-high performance liquid chromatograph (UPLC, Waters, USA). The Acquity BEH C_18_ column (1.7 μm, 2.1 mm × 100 mm) was used as an analytical column, and the detection wavelength was 250 nm. The mobile phase was consisted with methanol/water = 10/90 (v/v). The flow rate was 0.2 ml/min, and the injection volume was 3 μl.

The OXA@Fn solution (2 ml, 2 mg/ml) was transferred into a dialysis bag (with a molecular weight cut-off of 1000 Da), and then the dialysis bag was respectively immersed in 10 ml PBS with pH 7.4, 6.5, and 5.0. The OXA@Fn solution was dialyzed at 37 °C. 0.1 ml solution outside of dialysis bag was take out at 0.5, 1, 2, 4, 8, 12 and 24 h. Then the same volume of drug-free release medium was added into the dialysate outside of dialysis bag. the amount of OXA released from OXA@Fn at different time points was detected by UPLC.

### The uptake experiment

The total protein of GL261/TR cells, BV2 cells and mouse primary neurons were extracted. Western blot was used to detect the expression of TfR1 in GL261/TR cells, BV2 cells and mouse primary neurons.

GL261/TR cells, BV2 cells and mouse primary neurons were respectively inoculated into different 24-well plate with cover glass (2 × 10^5^ cells/well). 24 h after incubation, three cover glass planted with different cells were transferred to one cell culture dish. Cy5 labeled OXA@Fn (OXA@Fn-Cy5, 45 μg Fn/ml) was added into cell culture dish, and the cells were incubated for 0.5, 2 and 4 h. Cells on cover glass were fixed with 4% paraformaldehyde solution for 15 min. After that, the cell nucleus was stained with DAPI, and the uptake of OXA@Fn-Cy5 by GL261/TR cells, BV2 cells and mouse primary neurons was observed by laser scanning confocal microscope (LSCM, Nikon, A1Si, Japan).

GL261/TR cells were inoculated into 24-well plates with cover glass (2 × 10^5^ cells/well) and cultured for 24 h. Then, the cell culture medium was replaced with fresh cell culture medium containing TfR1 antibody. After incubation for 1 h, fresh cell culture medium containing OXA@Fn-Cy5 (45 μg Fn/ml) was added into culture medium, and cells were incubated for 4 h. (1) GL261/TR cells were collected and suspended in PBS. The uptake of OXA@Fn-Cy5 in GL261/TR cells was detected by flow cytometer (Beckman, CytoFlex3, USA). (2) GL261/TR cells were fixed with 4% paraformaldehyde solution for 15 min, and then the cell nucleus was stained with DAPI. The uptake of OXA@Fn-Cy5 in GL261/TR cells was observed by LSCM.

GL261/TR cells were inoculated into 24-well plates with cover glass (2 × 10^5^ cells/well) and cultured for 24 h. Then, the cell culture medium was replaced with fresh serum-free medium containing chlorpromazine (10 μg/ml), colchicine (800 μg/ml), methyl-β-cyclodextrin (5 μg/ml) and 2-deoxyd-glucose (900 μg/ml), respectively. Another group of GL261/TR cells were placed at 4 ℃. After 1 h, OXA@Fn-Cy5 (45 μg Fn/ml) was added, and cells were incubated for 4 h. (1) GL261/TR cells were collected and suspended in PBS. The uptake of OXA@Fn-Cy5 in GL261/TR cells was detected by flow cytometry. (2) GL261/TR cells were fixed with 4% paraformaldehyde solution for 15 min. After that, the cell nucleus was stained with DAPI, and the uptake of OXA@Fn-Cy5 in GL261/TR cells was detected by LSCM.

GL261/TR cells were inoculated into 24-well plates with cover glass (2 × 10^5^ cells/well) and cultured for 24 h. Then, the cell culture medium was replaced with fresh serum-free medium containing OXA@Fn-Cy5 (45 μg Fn/ml). After incubation for 0.5, 2 and 4 h, GL261/TR cells were fixed with 4% paraformaldehyde for 15 min. Next, TfR1 and lysosome in GL261/TR cells were respectively labeled with TfR1 antibody and lysosome antibody by using immunofluorescence method, and the cell nucleus was stained with DAPI. The distribution of OXA@Fn-Cy5 in GL261/TR cells was observed by LSCM.

### MTT experiment

GL261/TR cells were inoculated into 96-well plates (6 × 10^3^ cells/well) and cultured for 24 h. Then, the cell culture medium was replaced with fresh serum-free medium containing Fn, OXA, OXA@Fn, OXA@Fn+DFO, OXA@Fn+Fer-1 and OXA@Fn+IFN-γ. The equivalent OXA concentration was 0.5, 1, 3, 10 and 50 μg/ml. The concentration of DFO, Fer-1 and IFN-γ was 60 μg/ml, 3 μg/ml and 10 ng/ml, respectively. After cells were incubated for 48 h. MTT solution (20 μl, 5 μg/ml) was added into each well, and cells were incubated for 4 h. Then the cell culture medium was discarded. Dimethylsulfoxide (DMSO, 150 μl) was added into each well, and the absorbance of each well was detected at 492 nm by Molecular device (CMax Plus, shanghai, China). The cell survival rate was calculated.

### Live and dead cell staining experiment

GL261/TR cells were inoculated into 6-well plates (1 × 10^6^ cells/well) and cultured for 24 h. Then, the cell culture medium was replaced with fresh serum-free medium containing OXA, OXA@Fn, OXA@Fn+DFO, OXA@Fn+Fer-1 and OXA@Fn+IFN-γ. The equivalent OXA concentration was 3 μg/ml. The concentration of DFO, Fer-1 and IFN-γ was 60 μg/ml, 3 μg/ml and 10 ng/ml, respectively. GL261/TR cells were incubated for 48 h. After the cells were collected and stained with a live and dead cell staining kit, the number of live and dead cells was observed by fluorescence microscopy (Nikon, TS2R, Japan), and the ratio of live and dead cells was calculated.

### Cell cloning formation experiment

GL261/TR cells were inoculated into 6-well plates (500 cells/well) and cultured for 24 h. Then, the cell culture medium was replaced with fresh serum-free medium containing OXA, OXA@Fn, OXA@Fn+DFO, OXA@Fn+Fer-1 and OXA@Fn+IFN-γ. The equivalent OXA concentration was 3 μg/ml. The concentration of DFO, Fer-1 and IFN-γ was 60 μg/ml, 3 μg/ml and 10 ng/ml, respectively. GL261/TR cells were incubated for 24 h. The culture medium was replaced with DMEM complete culture medium containing serum, and the culture medium was changed every 2 days until visible cell clones were grown. After staining with 0.1% crystal violet solution, the cell clones were photographed and counted.

### Detection GSH, Fe^2+^ and H_2_O_2_ level in GL261/TR cells

GL261/TR cells were inoculated into 6-well plates (1 × 10^6^ cells/well) and cultured for 24 h. Then, the cell culture medium was replaced with fresh serum-free medium containing Fn (45 μg/ml), OXA, OXA@Fn, OXA@Fn+DFO, OXA@Fn+Fer-1 and OXA@Fn+IFN-γ. The equivalent OXA concentration was 3 μg/ml. The concentration of DFO, Fer-1 and IFN-γ was 60 μg/ml, 3 μg/ml and 10 ng/ml, respectively. GL261/TR cells were incubated for 24 h. (1) GL261/TR cells were collected and GSH content in cell was detected by using GSH detection kit (Solarbio, Beijing, China) [29]. Briefly, the collected GL261/TR cells were mixed with 1 ml reagent 1, and the cells were broken by ultrasound in ice bath. The above mixture was centrifuged for 10 min (8000×*g*, 4 °C). 200 μl supernatant was mixed with 100 μl reagent 2 and 40 μl reagent 3 thoroughly. After the mixture was left for 2 min at 37 °C, the absorbance of mixture was determined at 412 nm. The GSH concentration was calculated according to the standard curve. (2) GL261/TR cells were collected and Fe^2+^ content in cell was detected by using Fe^2+^ detection kit (Solarbio, Beijing, China) [30]. Briefly, the collected GL261/TR cells were mixed with 500 μl reagent 1, and the cells were broken by ultrasound in ice bath. The above mixture was centrifuged for 10 min (10,000×*g*, 4 °C). 200 μl supernatant was mixed with 100 μl reagent 2 thoroughly. After the mixture was left for 10 min at 37 °C, 100 μl chloroform was added, and the mixture was vibrated for 5 min. Then the mixture was centrifuged for 10 min (12,000×*g*, 25 °C), and the absorbance of water phase was determined at 593 nm. The Fe^2+^ concentration was calculated according to the standard curve. (3) GL261/TR cells were collected and H_2_O_2_ content in cell was detected by using H_2_O_2_ detection kit (Solarbio, Beijing, China) [31]. Briefly, the collected GL261/TR cells were mixed with 1 ml reagent 1, and the cells were broken by ultrasound in ice bath. The above mixture was centrifuged for 10 min (8000×*g*, 4 °C). The whole supernatant was mixed with 100 μl reagent 2 and 200 μl reagent 3 thoroughly. Then the mixture was centrifuged for 10 min (4000×*g*, 25 °C), and the precipitation was dissolved into 1 ml reagent 4. After the solution was left for 5 min at 37 °C, the absorbance of solution was determined at 415 nm. The H_2_O_2_ concentration was calculated according to the standard curve.

### Detection reactive oxygen species (ROS) and lipid peroxidation (LPO) level in GL261/TR cells

GL261/TR cells were inoculated into 24-well plates (2 × 10^5^ cells/well) with cover glass and cultured for 24 h. Then, the cell culture medium was replaced with fresh serum-free medium containing Fn (45 μg/ml), OXA, OXA@Fn, OXA@Fn+DFO, OXA@Fn+Fer-1 and OXA@Fn+IFN-γ. The equivalent OXA concentration was 3 μg/ml. The concentration of DFO, Fer-1 and IFN-γ was 60 μg/ml, 3 μg/ml and 10 ng/ml, respectively. GL261/TR cells were incubated for 24 h. For ROS level detection, 2,7-dichlorodihydrofluorescein diacetate (DCFH-DA, 10 μM) diluted with serum-free DMEM culture medium was added into cell culture medium, and cells were incubated for 20 min. After that, cells were fixed with 4% paraformaldehyde, and cell nucleus was stained with DAPI. ROS level in GL261/TR cells was detected by LSCM. For LPO level detection, boron difluoride pyrrole fluorescent dyes-C11 (C11-BODIPY, 1 μM) diluted with serum-free DMEM culture solution was added into cell culture medium, and cells were incubated for 20 min. After that, cells were fixed with 4% paraformaldehyde solution, and cell nucleus was stained with DAPI. LPO level in GL261/TR cells were detected by LSCM.

### The expression of GPX4 in GL261/TR cell

GL261/TR cells were inoculated into 6-well plates (1 × 10^6^ cells/well) and cultured for 24 h. Then, the cell culture medium was replaced with fresh serum-free medium containing OXA, OXA@Fn, OXA@Fn+DFO, OXA@Fn+Fer-1 and OXA@Fn+IFN-γ. The equivalent OXA concentration was 3 μg/ml. The concentration of DFO, Fer-1 and IFN-γ was 60 μg/ml, 3 μg/ml and 10 ng/ml, respectively. GL261/TR cells were incubated for 24 h. Cells were collected, and the proteins were extracted and quantified by BCA method. The expressions of GPX4 in GL261/TR cells were detected by western blot.

### Migration and invasion experiment

For migration experiment, GL261/TR cells were inoculated into transwell donor chamber (8 × 10^4^ cells/well) and cultured for 4 h. For invasion experiment, the donor chamber of transwell was precoated with Matrigel, and GL261/TR cells were inoculated into donor chamber (8 × 10^4^ cells/well). GL261/TR cells were cultured for 4 h. The cell culture medium in donor chamber was replaced with fresh serum-free medium containing OXA (1 and 3 μg/ml) and OXA@Fn (1 and 3 μg OXA/ml), and cells were incubated for 24 h. The donor chamber and recipient chamber were separated, and the cells inside the donor chamber were wiped with a cotton swab. The cells outside the donor chamber were fixed with 4% paraformaldehyde and stained with 0.1% crystal violet solution. The cells outside the donor chamber were observed by fluorescence microscopy. 1 ml of glacial acetic acid solution (33%) was added into donor chamber, the absorbance of glacial acetic acid solution in donor chamber was detected at 570 nm by Molecular device. The migration and invasion rate were calculated, respectively.

### The expression of invasion-related protein in GL261/TR cell

GL261/TR cells were inoculated into 6-well plates (1 × 10^6^ cells/well) and cultured for 24 h. Then, the cell culture medium was replaced with fresh serum-free medium containing OXA (3 μg/ml) and OXA@Fn (3 μg OXA/ml), and cells were incubated for 24 h. Cells were collected, and the proteins were extracted and quantified by BCA method. The expression of E-cadherin, N-cadherin, TGF-β, MMP-9 and CD44 in GL261/TR cells were detected by western blot.

### The in vitro DC cell maturation experiment

The femur and tibia of C57 mice were separated. DC cells in the bone marrow cavity were extracted and inoculated into a 6-well plate (3 × 10^6^ cells/well). Three days later, 1 ml of RPMI1640 containing GM-CSF (20 ng/ml) was added into culture medium, and the DC cells was cultured for 5 days.

GL261/TR cells were inoculated in donor chamber (7.5 × 10^4^ cells/well) and cultured for 4 h. OXA, OXA@Fn, OXA@Fn+DFO, OXA@Fn+Fer-1 and OXA@Fn+IFN-γ were added into donor chamber. The equivalent OXA concentration was 3 μg/ml. The concentration of DFO, Fer-1 and IFN-γ was 60 μg/ml, 3 μg/ml and 10 ng/ml, respectively. GL261/TR cells were incubated for 6 h. The donor chamber was then transferred into a 6-well plate inoculated with DC cells, and cells were cultured for 24 h. Cells in recipient chamber were collected and labeled with CD11c-APC, CD80-FITC and CD86-PE antibodies, the proportion of CD11c^+^CD86^+^CD80^+^ cells was detected by flow cytometer.

GL261/TR cells were inoculated into a 24-well plate with cover glass (1 × 10^5^ cells/well) and cultured for 24 h. Then, the cell culture medium was replaced with fresh serum-free medium containing OXA, OXA@Fn, OXA@Fn+DFO, OXA@Fn+Fer-1 and OXA@Fn+IFN-γ. The equivalent OXA concentration was 3 μg/ml. The concentration of DFO, Fer-1 and IFN-γ was 60 μg/ml, 3 μg/ml and 10 ng/ml, respectively. GL261/TR cells were incubated for 24 h. The cell culture medium was collected, and HMGB1 and ATP level in cell culture medium were detected by HMGB1 and ATP detection kit, respectively. At the same time, cells were fixed with 4% paraformaldehyde solution. CRT was labeled with CRT antibody by using immunofluorescence method, and the cell nucleus was stained with DAPI. The exposure of CRT on GL261/TR cell surface was observed by LSCM.

### The transport of OXA@Fn across in-vitro BBB

The in-vitro BBB model was established according to the literature [32]. When the TEER value was greater than 200 Ω cm^2^, the establishment of the in-vitro BBB model was considered successful.

The BBB model was transferred to a 12-well plate inoculated with GL261/TR cells (3 × 10^5^ cells/well), and OXA@Fn-Cy5 (45 μg Fn/ml) was added to the donor chamber. After incubation for 1, 2, 4 and 8 h, the TEER value was re-determined to determine the integrity of the in-vitro BBB model. The fluorescence intensity in the medium in the recipient chamber was measured by fluorescence spectrophotometer, and the transport efficiency of OXA@Fn-Cy5 across in-vitro BBB was calculated. GL261/TR cells from the receipt chamber were collected and the uptake of OXA@Fn-Cy5 in GL261/TR cells was detected by flow cytometer and LSCM.

### Establishment of orthotopic TMZ-resistant glioma model in mice

The orthotopic TMZ-resistant glioma model in mice was established by planting GL261/TR cells in brain tissue according to the literature [33]. Briefly, luciferase labeled GL261/TR cells (1 × 10^8^ cells/ml, 5 μl) were injected into the white matter of brain in C57 mouse at the right front 2 mm of the intersection of sagittal suture and coronal suture. 2 min after the injection of GL261/TR cells, the needle was slowly removed, and the injection hole was closed with bone wax to produce orthotopic TMZ-resistant glioma mice model.

### The biodistribution of OXA@Fn in orthotopic TMZ-resistant glioma mice

On the 6th day after the plantation of GL261/TR cells, mice were intraperitoneally injected with luciferase substrate, and tumor growth was observed by IVIS Lumina X5 (Perkin Elmer, USA). Mice that tumor growth did not meet the requirements were eliminated.

On the 10th day after the establishment of orthotopic TMZ-resistant glioma model in mice, OXA@Fn-Cy5 (3 mg OXA/kg) was injected into mice by tail vein. At 2 h, 12 h and 24 h after injection, the distribution of OXA@Fn-Cy5 in mice was observed by in vivo imager, and the comprehensive brain target efficiency was calculated. Brain tissue was collected and fixed with 4% paraformaldehyde solution. After brain tissue was cut into 4 µm slides, the cell nucleus was labeled with DAPI, and the blood vessels were labeled CD31 antibody by using immunofluorescence method. The distribution of OXA@Fn-Cy5 in orthotopic TMZ-resistant glioma tissue was observed by LSCM. The content of Fe^2+^ in orthotopic TMZ-resistant glioma tissue was detected by using Fe^2+^ detection kit [30].

### The therapeutic effect of OXA@Fn on orthotopic TMZ-resistant glioma in mice

On the 7th day after the establishment of the orthotopic TMZ-resistant glioma model, mice were injected with normal saline, OXA (3 mg/kg) and OXA@Fn (1.5, 3.0, 6.0 mg/kg) through tail vein, once every 3 days, for a total of 4 doses. The growth of orthotopic glioma was detected by in vivo imaging. The body weight and survival time of mice in each group were recorded, and the survival time curve was drawn using Prism 8.0. The mice were sacrificed on the second day after the last drug administration, and the brain tissue was removed. After the paraformaldehyde fixed brain tissue was cut into 4 µm slides, Ki67, Tunel, H&E and γ-H2AX staining were performed to observe the DNA damage, apoptosis and proliferation of orthotopic TMZ-resistant glioma. The expression of RCT and GPX4 in the slides were labeled by using immunofluorescence method. Dihydroethidium (DHE) probe was used to stain ROS in frozen sections of orthotopic TMZ-resistant glioma tissues. The expression of GPX4, xCT, HMGB1, RCT, cleaved caspase-3, Bax, Bcl-2, MMP-9, CD44, TGF-β, E-cadherin and N-cadherin in orthotopic TMZ-resistant glioma tissue were detected by western blot.

### The effect of OXA@Fn on immune microenvironment in orthotopic TMZ-resistant glioma in mice

On the 7th day after the establishment of the orthotopic TMZ-resistant glioma model, mice were injected with normal saline, OXA (3 mg/kg) and OXA@Fn (1.5, 3.0, 6.0 mg/kg) through tail vein, once every 3 days, for a total of 4 doses. The mice were sacrificed on the second day after the last drug administration, and the orthotopic glioma tissue was isolated, and its single-cell suspension was prepared. The number of CD11c^+^CD86^+^CD80^+^ cells (mDCs), Iba-1^+^CD86^+^ cells (M1 type GAM), Iba-1^+^CD206^+^ cells (M2 type GAM), CD4^+^CD25^+^FoxP3^+^ T cells (T_reg_ cells), and CD3^+^CD8^+^IFN-γ^+^ T cells (T_eff_ cells) were detected by flow cytometer. At the same time, the orthotopic glioma tissue was homogenized (1 ml homogenate contained 100 mg glioma tissue), and the level of HMGB1, ATP, IL-10, IL-12, TGF-α and IFN-γ in orthotopic glioma tissue were detected by ELISA kit. The orthotopic glioma tissue was fixed with 4% paraformaldehyde. After orthotopic glioma tissue was cut into 4 µm slides, the expressions of Iba-1, CD86 and CD206 in orthotopic glioma tissue were observed by immunofluorescence staining, and the proportion of Iba-1^+^CD86^+^ cells and Iba-1^+^CD206^+^ cells were calculated.

### The preliminary safety evaluation of OXA@Fn in mice

Normal mice were injected with normal saline, OXA (3.0, 6.0  mg/kg), OXA@Fn (1.5, 3.0, 6.0 mg/kg) through the tail vein, once every 3 days. After four consecutive doses, mice serum was obtained, and the activities of alanine aminotransferase (ALT) and aspartate aminotransferase (AST), as well as the contents of serum creatinine (CREA) and serum urea nitrogen (BUN) in serum were detected by automatic biochemical analyzer. The hematoxylin–eosin (H&E) staining of paraffin sections of brain, heart, liver, spleen, lung and kidney in each group were obtained, and the effect of OXA@Fn on tissue morphology was observed by inverted microscope.

### Statistical analysis

All data are expressed as mean ± SD and were analyzed by GraphPad Prism 8.0 software. The t test was used for comparison between the two groups, and one-way ANOVA was used for comparison between multiple groups. P < 0.05 was considered to be statistically significant between the two groups.

## Results and discussion

### The enhancement of OXA on the ferroptosis of GL261/TR cells caused by Fn

**Fig. 1:**
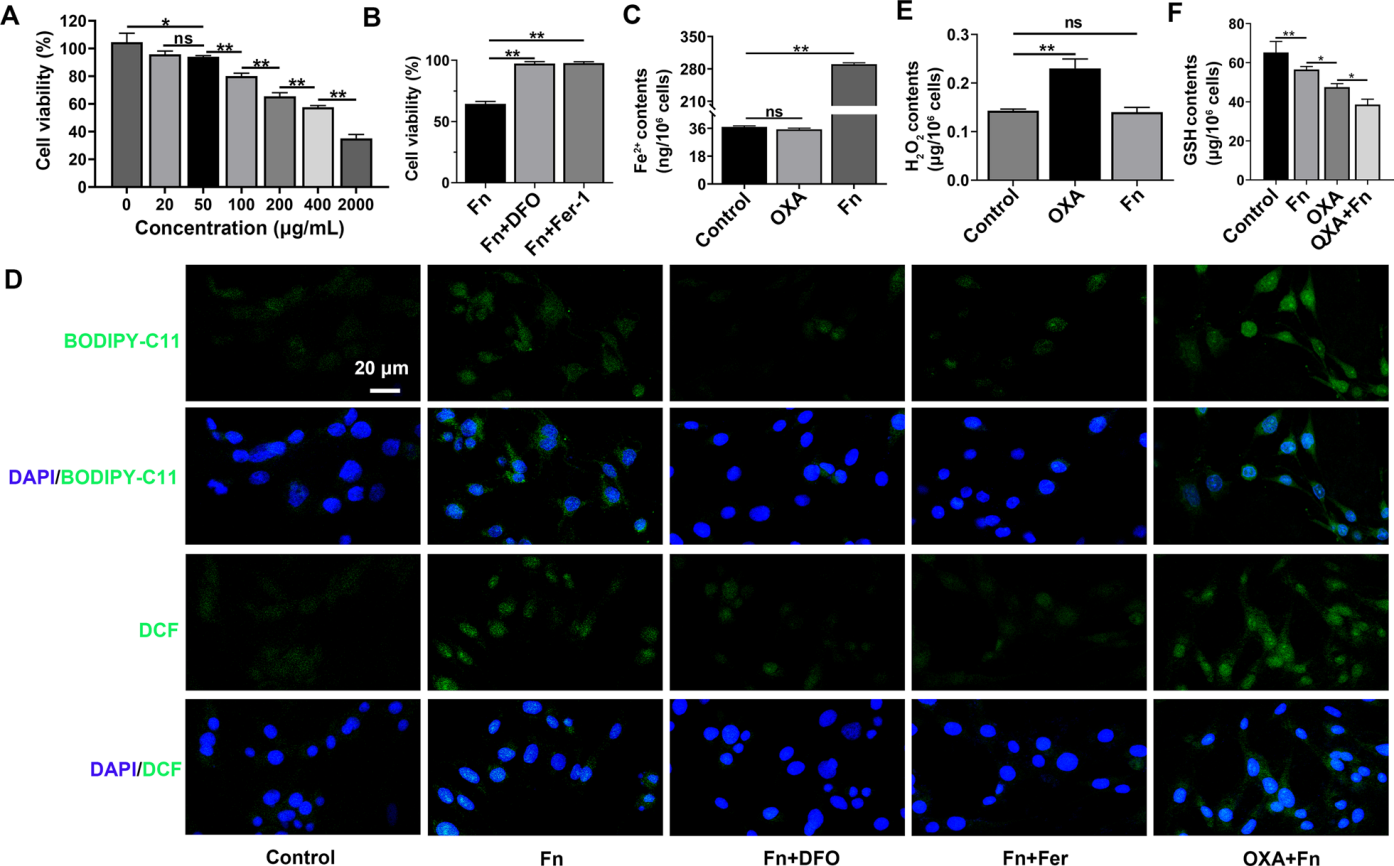
The effects of Fn and OXA on the activity of GL261/TR cells. **A** The effect of Fn on the proliferation of GL261/TR cells. **B** The effect of DFO (60 μg/ml) and Fer-1 (3 μg/ml) on the efficacy of Fn in GL261/TR cells. **C** The effect of Fn on Fe^2+^ level in GL261/TR cells. **D** The effects of Fn on LPO and ROS levels in GL261/TR cells. **E** The effect of OXA on H_2_O_2_ level in GL261/TR cells. **F** The effect of OXA and Fn on GSH level in GL261/TR cells. The Fn concentration was 200 μg/ml, and OXA concentration was 3 μg/ml. (n = 3, mean ± SD, ns: significant difference, *P < 0.05, **P < 0.01)

MTT assay indicated that Fn inhibited the proliferation of GL261/TR cells in dose-dependent manner. In the presence of DFO and Fer-1, the inhibitory effect of Fn on the proliferation of GL261/TR cells was attenuated (Fig. 1A, B). Fn increased Fe^2+^, ROS and LPO levels in GL261/TR cells. In the presence of DFO and Fer-1, the levels of ROS and LPO in GL261/TR cells were significantly reduced, indicating after Fn entered GL261/TR cells, Fe^2+^ was released from Fn, which induced the ferroptosis of GL261/TR cells (Fig. 1C, D). OXA can activate niacinamide adenine dinucleotide phosphate (NADPH) oxidase (NOXs), and activated NOXs can catalyze NADPH and oxygen (O_2_) to generate NADP^+^ and superoxide free radicals (O_2_^**.**−^). Then superoxide dismutase (SOD) catalyzes the disproportionation reaction of O_2_^**.**−^ to produce hydrogen peroxide (H_2_O_2_) [28, 34]. The experimental results indicated that OXA significantly increased the level of H_2_O_2_ in GL261/TR cells (Fig. 1E). As compared with OXA, the GSH level was further decreased when OXA was used with Fn (Fig. 1F). When OXA was used with Fn, the ROS and LPO level in GL261/TR cells was further increased, and the inhibitory effect of OXA on the proliferation of GL261/TR cells was markedly enhanced, suggesting after exogenous Fn was taken up by GL261/TR cells, Fn was degraded in the lysosomes, resulting in the release of Fe^2+^, which can react with H_2_O_2_ induced by OXA to produce hydroxyl free radicals, and eventually lead to the enhancement of ferroptosis of GL261/TR cells [35, 36]. These results demonstrated that when OXA was used with Fn, the apoptosis and ferroptosis of GL261/TR cells was mutually boosted, which synergistically inhibited the proliferation of GL261/TR cells. It has been demonstrated that ferroptosis can activates effector T cell (T_eff_ cell) and promotes T cell mediated anti-tumor immune response [23]. This suggested when OXA was used with Fn to treat TMZ-resistant glioma, T cell mediated anti-tumor immune response induced by ferroptosis would be enhanced in the body.

### Characterization of OXA@Fn

**Fig. 2:**
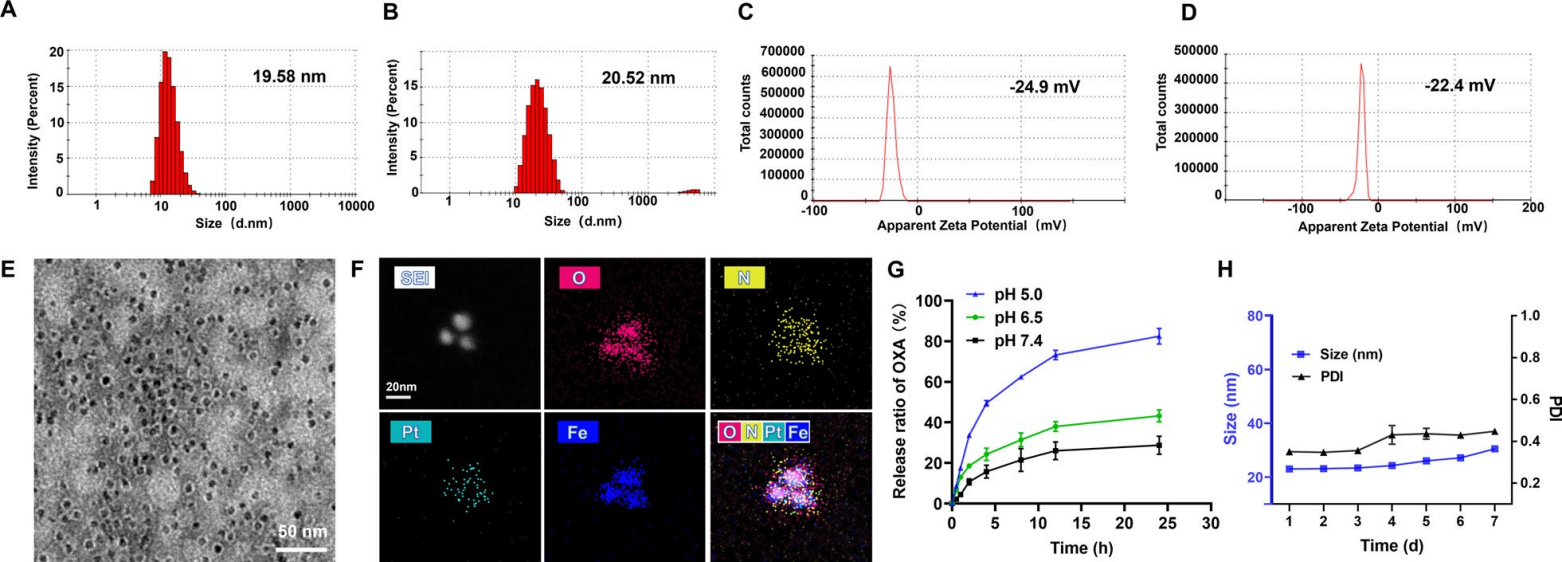
Characterization of OXA@Fn. **A**, **B** Particle size distribution of Fn and OXA@Fn. **C**, **D** Zeta potential distribution of Fn and OXA@Fn. **E** TEM image of OXA@Fn. **F** Element mapping analysis diagram of OXA@Fn. **G** In vitro OXA release from OXA@Fn in different pH release medium. **H** Stability of OXA@Fn in PBS. (n = 3, mean ± SD)

The particle size and zeta potential of OXA@Fn were 20.52 nm and − 22.4 mV, respectively. As compared with Fn, the particle size and zeta potential of OXA@Fn were not significantly changed, indicating that the structure of Fn was not influenced after encapsulating OXA in Fn (Fig. 2A–D). OXA@Fn was spherical with uniform particle size (Fig. 2E). Element mapping analysis showed that OXA@Fn contained O, N, Pt and Fe elements (Fig. 2F), and XPS results showed that the percentage content of C, O, N, Pt and Fe element in OXA@Fn was 59.72%, 27.97%, 9.61%, 1.95% and 0.75%, respectively (Additional file 1: Fig. S1). The above data indicated that OXA was successfully encapsulated in Fn. The drug loading of OXA in OXA@Fn was 6.3% and the encapsulation efficiency was 21%. About 28.8%, 43% and 82% of OXA were released from OXA@Fn within 24 h in pH 7.4, 6.5 and 5.0 release medium, respectively (Fig. 2G). These results indicated that Fn could be depolymerized in acidic environment such as in lysosome and promoted the release of OXA from OXA@Fn [37, 38]. The particle size of OXA@Fn in PBS did not change significantly within 7 days (Fig. 2H), suggesting that OXA@Fn had good stability in vitro.

### The uptake mechanism of OXA@Fn by GL261/TR cells

**Fig. 3:**
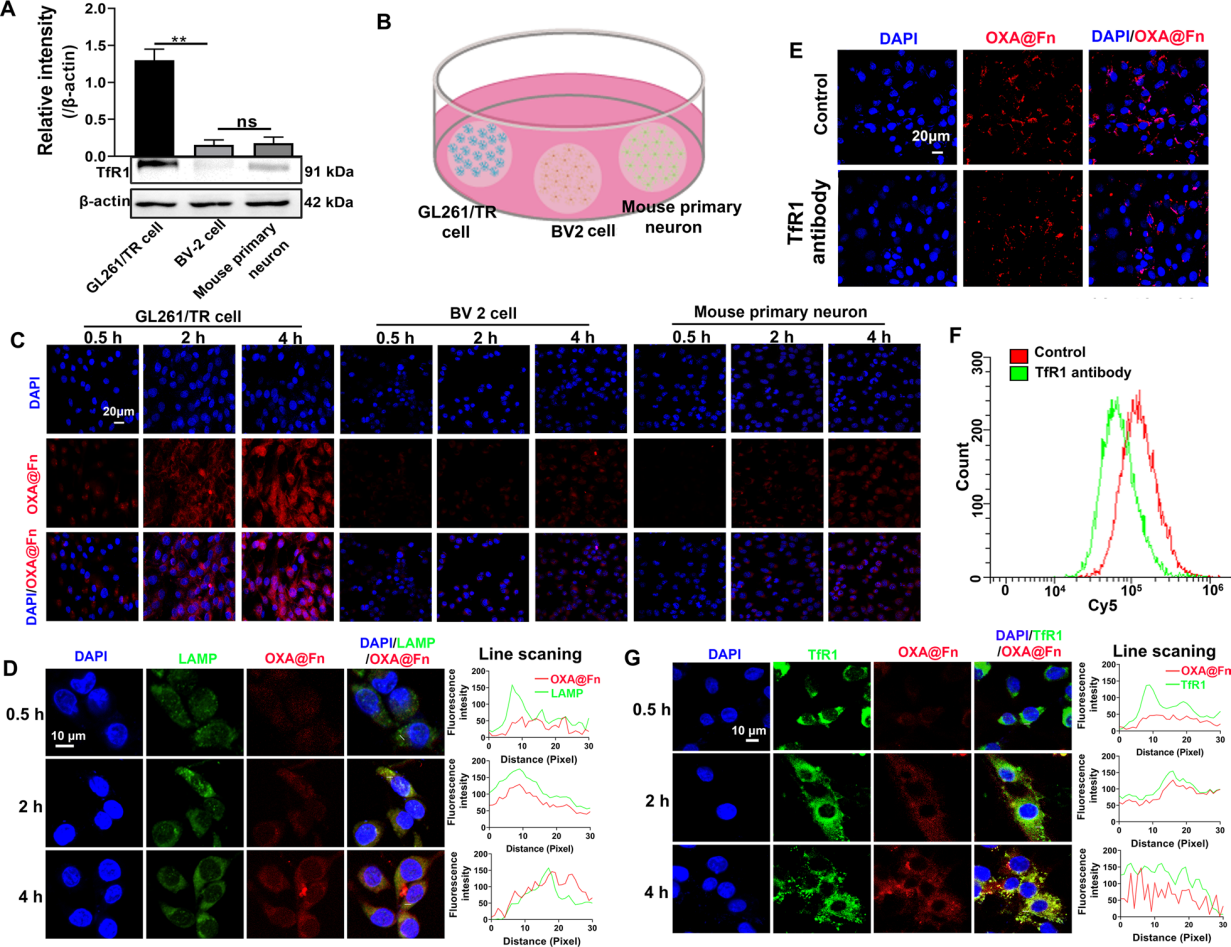
The uptake of OXA@Fn by GL261/TR cell. **A** The expression of TfR1 in GL261/TR cells, BV2 cells and mouse primary neurons. **B** Schematic illustrations of co-culture GL261/TR cells, BV2 cells and mouse primary neurons in one cell culture dish. **C** Uptake of OXA@Fn by co-cultured GL261/TR cells, BV2 cells and mouse primary neurons. **D** The co-localization of OXA@Fn (red) and lysosome (green) in GL261/TR cells. **E** The uptake of OXA@Fn by GL261/TR cells in the presence of TfR1 antibody. **F** The typical flow cytometer diagrams of the uptake of OXA@Fn by GL261/TR cells in the presence of TfR1 antibody. **G** The co-localization of OXA@Fn (red) and TfR1 (green) in GL261/TR cells. The OXA@Fn concentration was equivalent to 45 μg Fn/ml. (n = 3, mean ± SD, ns: significant difference, **P < 0.01)

Studies have shown that Fn is taken up by the cell through recognizing and binding with transferrin receptor 1 (TfR1) on the cell surface [19, 39]. Therefore, the expression of TfR1 in GL261/TR cells, BV2 cells and primary mouse neurons was investigated by western blot. The results showed that the expression of TfR1 in GL261/TR cells was significantly higher than that in BV2 cells and primary mouse neurons, and a few amounts of TfR1 was expressed in BV2 cells and primary mouse neurons (Fig. 3A).

GL261/TR cells, BV2 cells and primary mouse neurons, planted in different cover glass previously, were co-cultured in one cell culture dish to investigate the uptake of OXA@Fn in different cells (Fig. 3B). The results indicated that GL261/TR cells took up OXA@Fn in time-dependent manner, while little amount of OXA@Fn was taken up by BV2 cells and primary mouse neurons (Fig. 3C). Further studies showed that OXA@Fn was enriched in lysosomes after being taken up by GL261/TR cells (Fig. 3D), which was conducive to the depolymerization of Fn and the release of OXA and Fe^2+^ to play a synergistic role.

When GL261/TR cells were pre-incubated with chlorpromazine and 2-deoxyglucose, the uptake of OXA@Fn by GL261/TR cells was significantly decreased. At 4 °C, the uptake of OXA@Fn by GL261/TR cells was attenuated (Additional file 1: Fig. S2). The above results indicated that the uptake of OXA@Fn by GL261/TR cell was mainly through clathrin-mediated endocytosis. When GL261/TR cells were pre-incubated with TfR1 antibody, the uptake of OXA@Fn by GL261/TR cells decreased significantly (Fig. 3E, F). In addition, when OXA@Fn was incubated with GL261/TR cells, a large amount of OXA@Fn was co-localized with TfR1 in GL261/TR cells (Fig. 3G). These data suggested that OXA@Fn was taken up through binding with TfR1 in GL261/TR cells. Moreover, the western blot results showed that expression of TfR1 in GL261/TR cells was significantly higher than that in BV2 cells and mouse primary neurons (Fig. 3A), which resulted in the accumulation of OXA@Fn in GL261/TR cells was significantly higher than that in BV2 cells and mouse primary neurons. This implies that OXA@Fn is able to specifically targeted to glioma cells when it reaches glioma tissue, and it would show little toxicity to neurons and microglia.

### The in vitro anti glioma activity of OXA@Fn

**Fig. 4:**
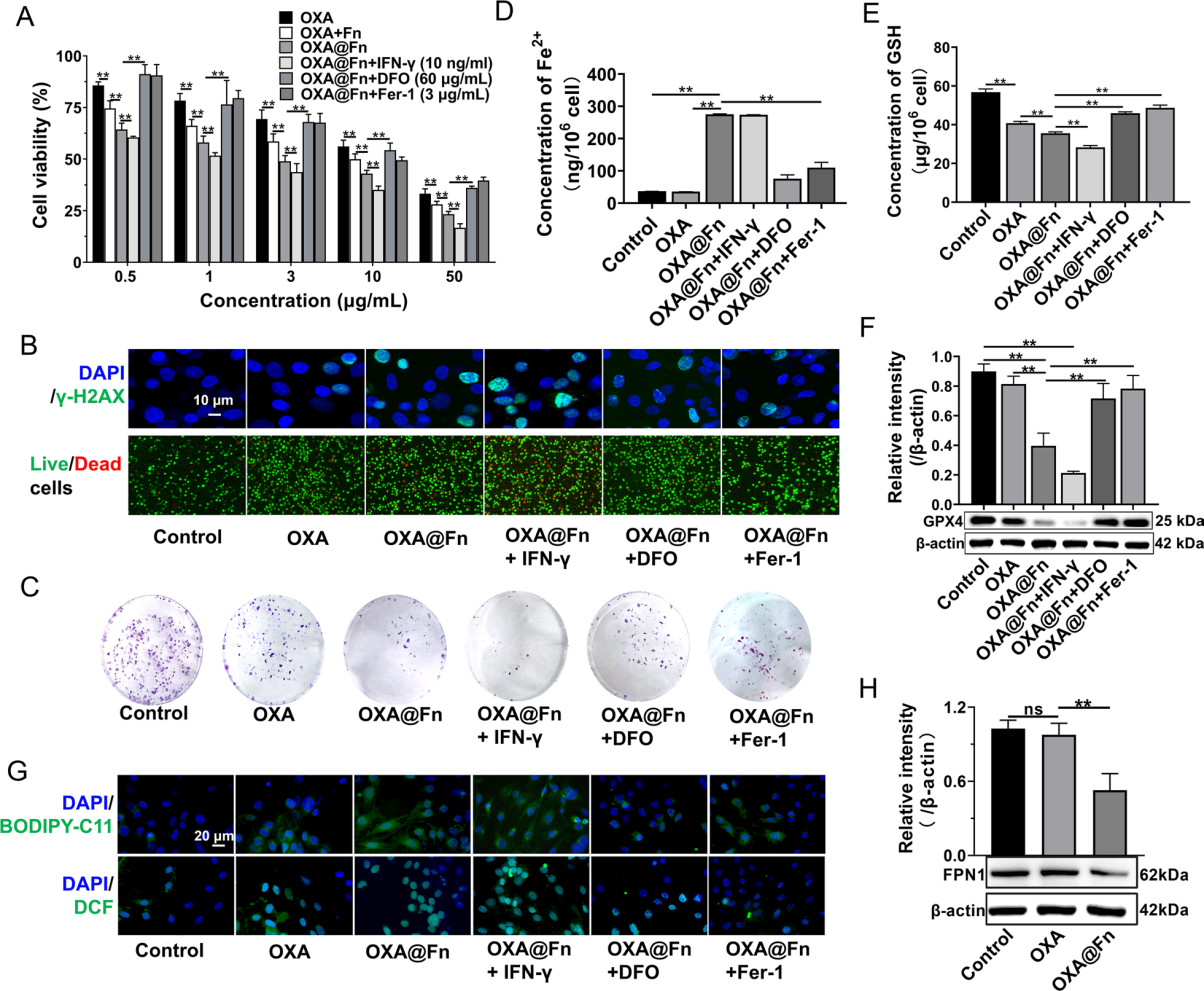
The ferroptosis induced by OXA@Fn in GL261/TR cells. **A** Effect of OXA@Fn on the viability of GL261/TR cells in the presence of IFN-γ (10 ng/ml), DFO (60 μg/ml) and Fer-1 (3 μg/ml). **B** The effect of OXA@Fn on DNA damage (marked with γ-H2AX antibody) and the live/death GL261/TR cells. **C** The effect of OXA@Fn on the cloning formation of GL261/TR cells. **D** The effect of OXA@Fn on Fe^2+^ level in GL261/TR cells. **E** The effect of OXA@Fn on GSH level in GL261/TR cells. **F** The effect of OXA@Fn on the expression of GPX4 in GL261/TR cells in the presence of IFN-γ (10 ng/ml), DFO (60 μg/ml) and Fer-1 (3 μg/ml). **G** The effect of OXA@Fn on LPO (marked with BODIPY-C11, green color) and ROS (marked with DCF, green color) level in GL261/TR cells. **H** The effect of OXA@Fn on the expression of FPN1 in GL261/TR cells. The OXA@Fn concentration was equivalent to 45 μg Fn/ml. (n = 3, mean ± SD, *P < 0.05, **P < 0.01)

OXA@Fn strongly inhibited the proliferation of GL261/TR cell and reduced the cloning formation of GL261/TR cell in a concentration-dependent manner as compared with OXA, indicating OXA@Fn has great potential in the treatment of TMZ-resistant glioma. In the presence of IFN-γ, the cytotoxicity of OXA@Fn on GL261/TR cells was enhanced. In the presence of DFO and Fer-1, the cytotoxicity of OXA@Fn on GL261/TR cells was reduced. It is reported that OXA can induce the damage of DNA, and γ-H2AX is a mark of the break of DNA double strand. The immunofluorescence results indicated that γ-H2AX level and the ratio of dead and live cell increased after the treatment of OXA@Fn. IFN-γ increased the level of γ-H2AX and cell death ratio in GL261/TR cells. DFO and Fer-1 reduced the level of γ-H2AX and cell death ratio in GL261/TR cells (Fig. 4A–C, Additional file 1: Fig. S3A, B), indicating the DNA damage and cytotoxicity caused by OXA@Fn was markedly attenuated in the present of inhibitor of ferroptosis (DFO or Fer-1). However, in the present of inducer of ferroptosis (IFN-γ), the DNA damage and cytotoxicity caused by OXA@Fn was markedly enhanced. The above data indicated that ferroptosis was involved in DNA damage and cytotoxicity caused by OXA@Fn. Moreover, Fe^2+^ level in GL261/TR cells significantly increased after administration of OXA@Fn (Fig. 4D), which resulted in a significant increase in LPO and ROS level in GL261/TR cells, while a significant decrease in GSH and GPX4 levels (Fig. 4E–G, Additional file 1: Fig. S3C–F). These data demonstrated that OXA@Fn significantly increased ferroptosis of GL261/TR cells, resulting in a large amount of GL261/TR cells death. Furthermore, it was reported that high intracellular GSH level could reduce the interaction between OXA and DNA in nucleus [40, 41]. The experimental results indicated that Fn significantly reduced the intracellular GSH level while increased LPO and ROS level in GL261/TR cells (Fig. 1D, F). Therefore, OXA@Fn greatly enhanced the interaction between OXA and DNA in nucleus, which resulted in the augmentation of DNA damage and ferroptosis, and subsequently boosted the cytotoxicity on GL261/TR cells.

Ferroportin 1 (FPN1), as the only known mammalian iron efflux membrane protein, is mainly responsible for transporting Fe^2+^ to the outside of the cell and maintaining Fe^2+^ homeostasis in the cell [42]. Studies have shown that increased intracellular ROS levels can inhibit the activity of NrF2 and further inhibit the expression of FPN1 [43, 44]. Therefore, we further investigated the effects of OXA@Fn on the expression of FPN1 in GL261/TR cells. The results showed that OXA@Fn significantly reduced the expression of FPN1 in GL261/TR cells (Fig. 4H), resulting in Fe^2+^ to be locked up inside the GL261/TR cells. This subsequently augmented the Fenton reaction and boosted ferroptosis of GL261/TR cells. These results suggested that OXA@Fn could not only induce DNA damage and apoptosis of GL261/TR cells but also boost ferroptosis of GL261/TR cells through hijacking Fe^2+^ and promoting the burst release of H_2_O_2_.

**Fig. 5:**
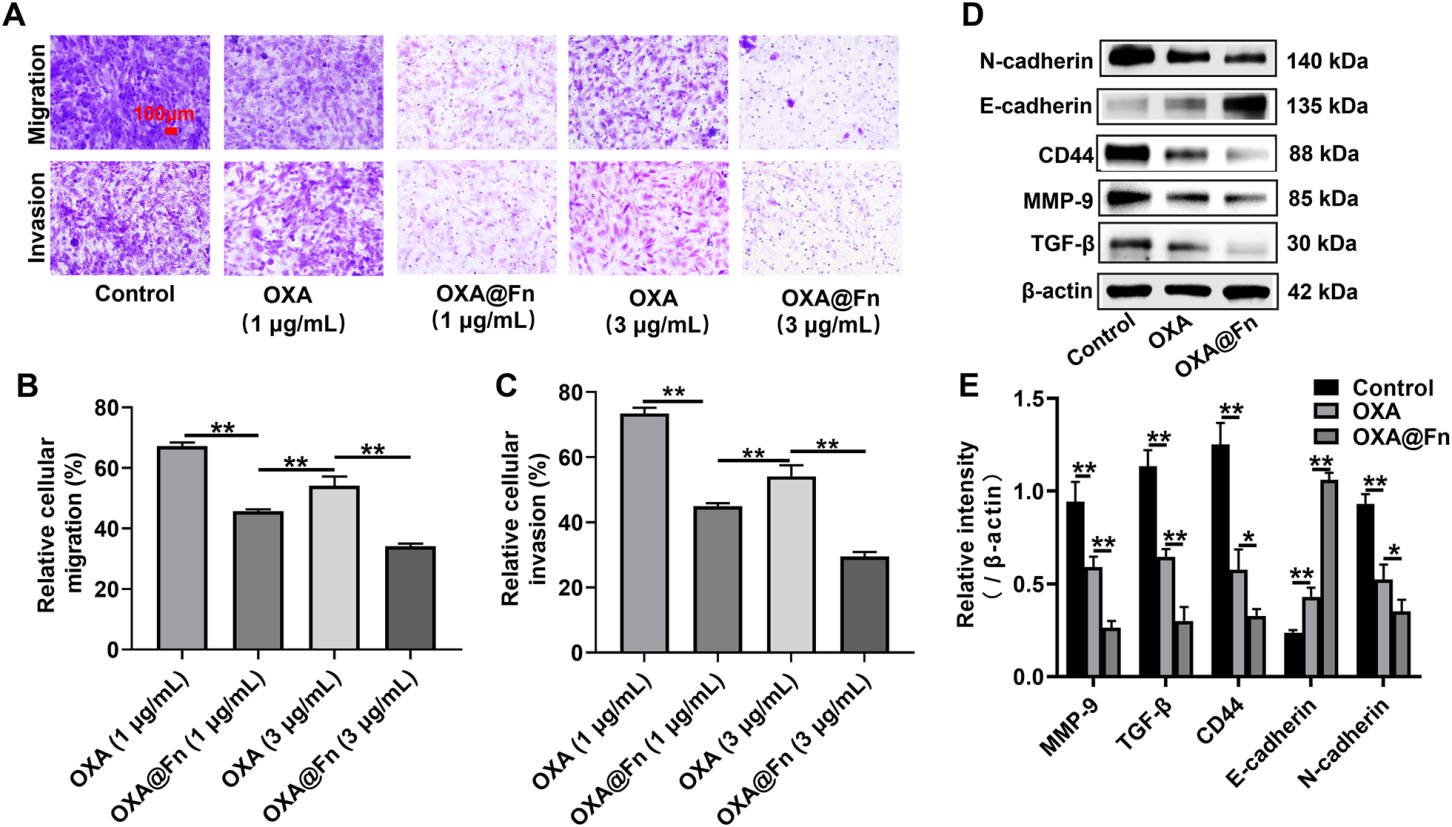
The effect of OXA@Fn on migration and invasion of GL261/TR cells. **A** The effect of OXA@Fn on the migration and invasion of GL261/TR cells. **B**, **C** The relative migration rate and invasion rate of GL261/TR cells after the treatment of OXA@Fn. **D** The effect of OXA@Fn on the expression of invasion-related proteins in GL261/TR cells. **E** Semi-quantitative statistic results of invasion-related protein. (n = 3, mean ± SD, *P < 0.05, **P < 0.01)

OXA@Fn inhibited the migration and invasion of GL261/TR cell in a concentration-dependent manner (Fig. 5A–C). Western blot showed that as compared with OXA, OXA@Fn significantly enhanced the expression of E-cadherin and decreased the expression of N-cadherin, TGF-β, MMP-9 and CD44 in GL261/TR cells (Fig. 5D, E). Studies have shown that E-cadherin is a key molecule in maintaining adhesion between cancer cells. Generally, the loss or decrease of E-cadherin expression in cancer cells is believed to be closely related to invasion and metastasis of cancer cells [45, 46]. In addition, cancer cells also can up-regulate N-cadherin, resulting in enhanced the motility of cancer cells. MMP-9 degrades E-cadherin, reduce adhesion between cancer cells, and promote cancer cell migration [45, 47]. Increased TGF-β levels can block the differentiation of immature T cells into Th1 cells, subsequently promote their transformation into T_reg_ and inhibit the antigen presenting function of DC cells, which subsequently lead to immune escape of cancer cells. Increased levels of TGF-β can also promote the proliferation and metastasis of cancer cells. CD44 is a cell adhesion molecule that promotes the proliferation and metastasis of cancer cells by increasing the expression of TGF-β [48]. The experimental results demonstrated that OXA@Fn decreased the migration and invasion of GL261/TR cells by promoting the expression of E-cadherin and inhibiting the expressions of N-cadherin, TGF-β, MMP-9 and CD44.

### The effect of OXA@Fn on DC cells maturation

**Fig. 6:**
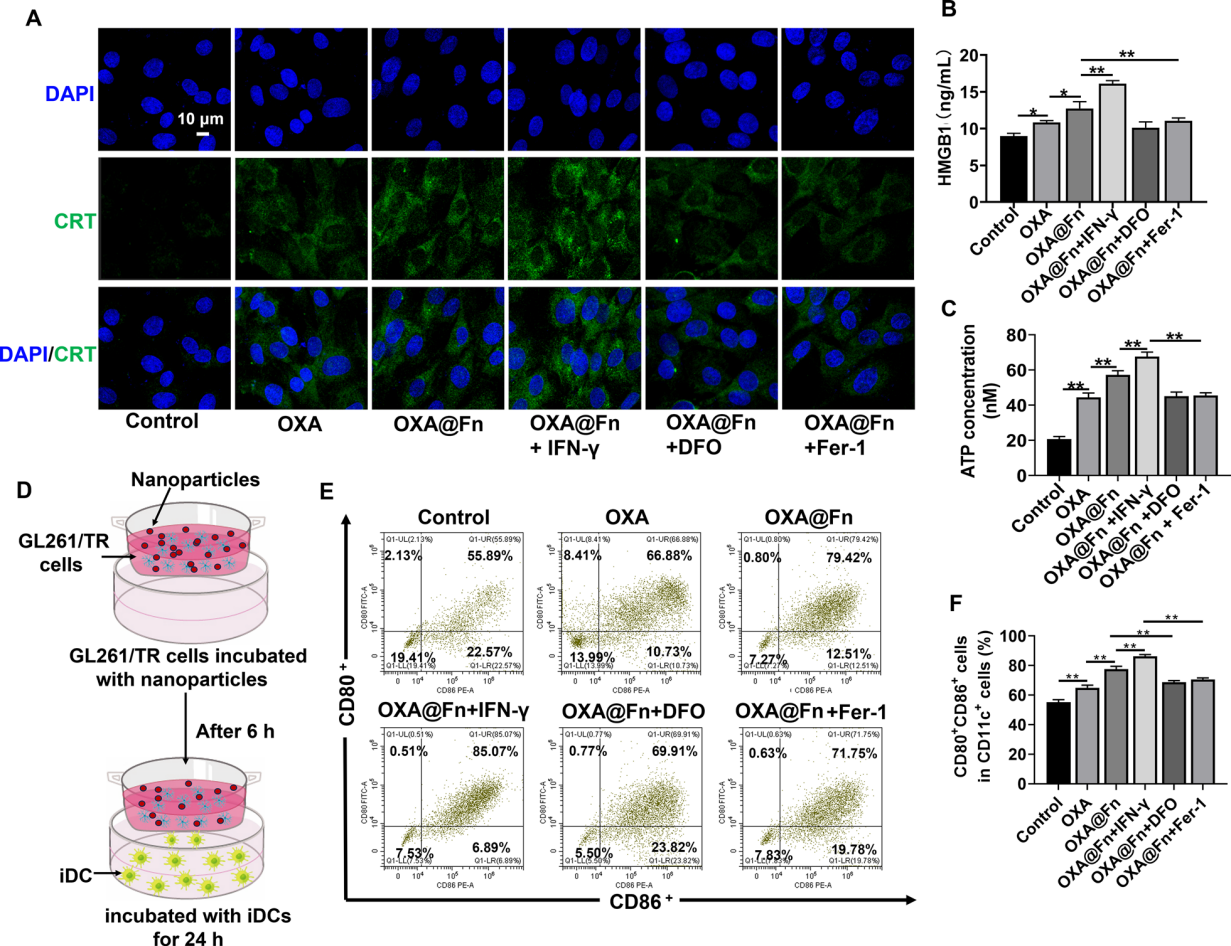
The effect of OXA@Fn on the maturation of DC cells in vitro. **A** CRT exposure in GL261/TR cells after the treatment OXA@Fn. **B**, **C** The secretion of HMGB1 and ATP from GL261/TR cells after the treatment OXA@Fn. **D** Schematic diagram of co-culture of GL261/TR cells and iDC cells. **E** The maturation of DC cells analyzed by flow cytometer. **F** Statistic analysis of the matured DC cells. The OXA@Fn concentration was equivalent to 45 μg Fn/ml. (n = 3, mean ± SD, *P < 0.05, **P < 0.01)

Cancer cells that undergo ferroptosis release a variety of immunostimulatory signals such as HMGB1, CRT and ATP. Immunofluorescence staining results showed that CRT exposure was increased after GL261/TR cells were treated with OXA@Fn, while CRT exposure was decreased when GL261/TR cells were pretreated with DFO and Fer-1. When GL261/TR cells were pretreated with IFN-γ, CRT exposure was increased (Fig. 6A). In addition, OXA@Fn increased the release of ATP and HMGB1 from GL261/TR cells (Fig. 6B, C). The above data indicated that ferroptosis was involved in the exposure of CRT and the release of HMGB1 and ATP from GL261/TR cells. OXA@Fn increased the exposure of CRT and the release of HMGB1 and ATP by inducing the ferroptosis of GL261/TR cells. Moreover, it is reported that ATP released by cancer cells can recruit DC cells into cancer tissue. CRT exposed on the surface of cancer cells can stimulate DC cells to phagocytosis of cancer cells. In addition, HMGB1 secreted by cancer cells can also promote the maturation of DC cells, and eventually induce specific T-cell mediated anti-tumor immunity [22, 32]. Thus, the effect of OXA@Fn on DC cells maturation was investigated in vitro. Flow cytometer results indicated that after the treatment of OXA@Fn, the proportion of CD11c^+^CD80^+^CD86^+^ cell was 79.42%, which was higher than that in control group and free OXA group. Moreover, after pretreatment with DFO and Fer-1, the proportion of CD11c^+^CD80^+^CD86^+^ cell was decreased. After pretreatment with IFN-γ, the proportion of CD11c^+^CD80^+^CD86^+^ cell was increased (Fig. 6D–F). These results demonstrated that OXA@Fn promoted the maturation of DC cells by inducing the ferroptosis of GL261/TR cells.

### Transport of OXA@Fn across BBB

**Fig. 7:**
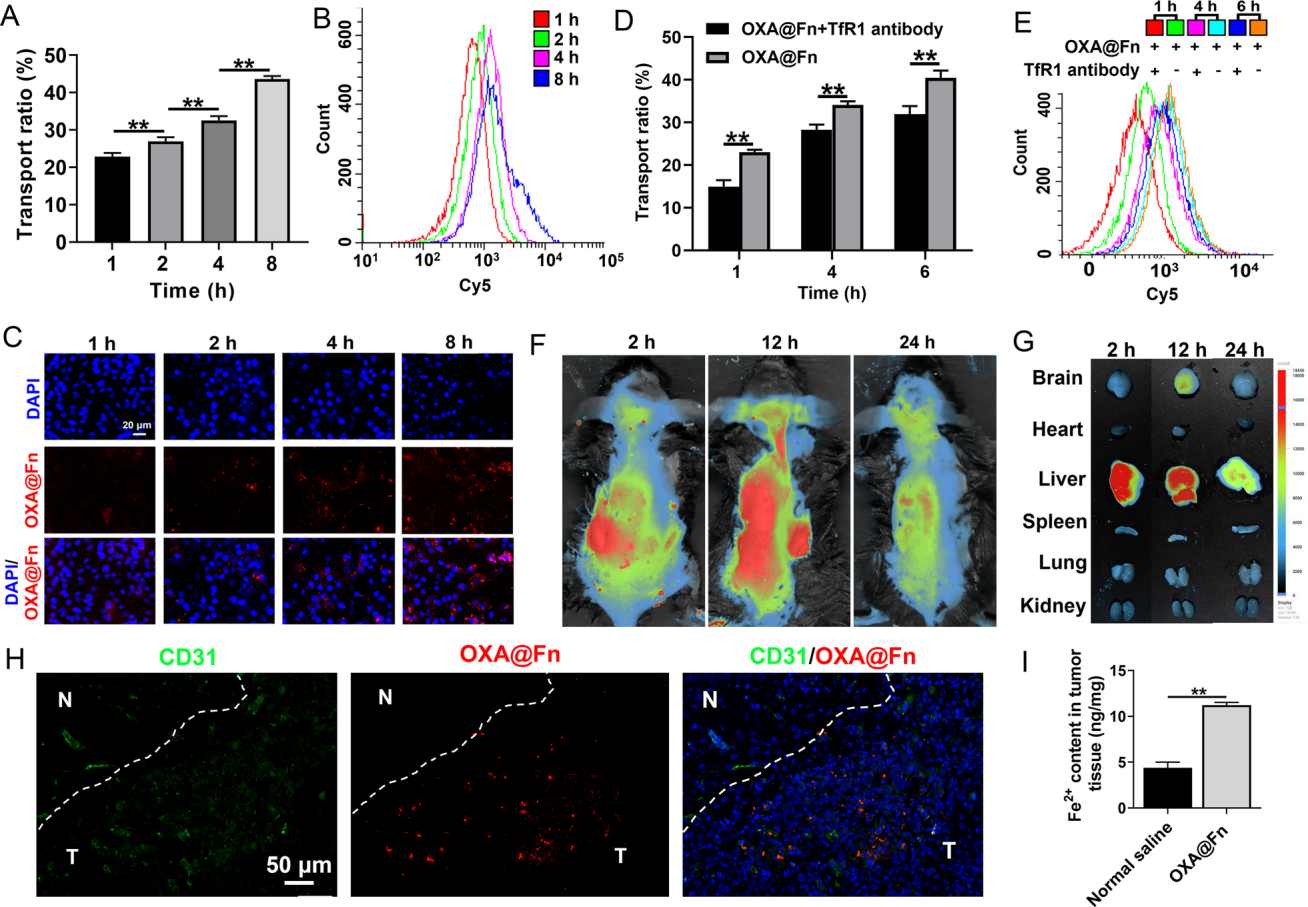
Transportation of OXA@Fn across BBB. **A** Transportation ratio of OXA@Fn across in vitro BBB detected by fluorescence spectrophotometry. **B**, **C** The accumulation of OXA@Fn in GL261/TR cells after penetrating in-vitro BBB detected by flow cytometer and LSCM. **D** The effect of TfR1 antibody on transportation ratio of OXA@Fn across the in-vitro BBB detected by fluorescence spectrophotometry. **E** The effect of TfR1 antibody on the accumulation of OXA@Fn in GL261/TR cells after penetrating in-vitro BBB detected by flow cytometry. **F** The distribution of OXA@Fn in orthotopic TMZ-resistant glioma mice observed by in vivo bioluminescence imaging. **G** The distribution of OXA@Fn in main organs of orthotopic TMZ-resistant glioma mice observed by in vivo bioluminescence imaging. **H** Distribution of OXA@Fn in orthotopic TMZ-resistant glioma tissue observed by CLSM. CD31 staining: green (stands for blood vessel). Cy5 staining: red (stands for OXA@Fn). T: tumor tissue; N: normal tissue. **I** The content of Fe^2+^ in orthotopic TMZ-resistant glioma tissue. The OXA@Fn concentration was equivalent to 45 μg Fn/ml in cell experiment. The dose of OXA@Fn was equivalent to 3 mg OXA/kg in animal experiment. (n = 3, mean ± SD, **P < 0.01)

After the treatment of OXA@Fn-Cy5, there was no significant change in electrical resistance between transwell donor chamber and recipient chamber, indicating that OXA@Fn-Cy5 did not damage the integrity of the in-vitro BBB model (Additional file 1: Fig. S4). The fluorescence spectrophotometry results showed that the concentration of OXA@Fn-Cy5 in recipient chamber increased with the extension of incubation time (Fig. 7A). At the same time, the uptake of OXA@Fn-Cy5 by GL261/TR cells in recipient chamber also augmented in time-dependent manner (Fig. 7B, C). In addition, western blot indicated that TfR1 was highly expressed in bEnd 3 cells (Additional file 1: Fig. S5). The concentration of OXA@Fn-Cy5 in recipient chamber and the accumulation of OXA@Fn in GL261/TR cells in recipient chamber decreased when TfR1 antibody was added to donor chamber (Additional file 1: Fig. 7D, E). The above results indicated that OXA@Fn-Cy5 crossed in-vitro BBB by binding with TfR1 on the surface of bEnd3 cells.

After injection of OXA@Fn-Cy5 by tail vein, the distribution of OXA@Fn-Cy5 in glioma mice was observed by vivo imager. The results showed that a certain amount of OXA@Fn-Cy5 distributed in the brain of glioma mice (Fig. 7F, G). The comprehensive brain targeting efficiency of OXA@Fn-Cy5 at 2 h, 12 h and 24 h was 11.28%, 23.80% and 16.77%, respectively (Additional file 1: Fig. S6). The results of LSCM indicated that OXA@Fn-Cy5 could permeate out of blood vessels in orthotopic TMZ-resistant glioma tissues. Most amount of OXA@Fn-Cy5 was distributed in orthotopic TMZ-resistant glioma tissue, while OXA@Fn-Cy5 was rarely distributed in normal brain tissue (Fig. 7H). Moreover, OXA@Fn increased Fe^2+^ content in orthotopic TMZ-resistant glioma tissues (Fig. 7I), indicating that OXA@Fn could not only accumulate in orthotopic TMZ-resistant glioma tissues but also could release Fe^2+^ in orthotopic TMZ-resistant glioma tissues.

### Anti orthotopic TMZ-resistant glioma activity of OXA@Fn in mice

Animal models of glioma include induced model, glioma cell transplantation model and transgenic model. The chemically-induced glioma model is produced by implanting tumorigenic substances such as methylcholanthrene and alkylating agents into the brain of mice. This model is not very similar to human glioma, so it is rarely used at present. The virus induced glioma model is created by injecting Rous sarcoma virus or adenovirus into the brain of mice. The virus induced glioma model can reflect the development of glioma, but the tumor formation ratio is low [49]. The orthotopic planted glioma model is made by implanting glioma cell into brain tissue to induce tumor formation. The orthotopic planted glioma model has advantage of high tumor formation ratio, simple operation and easy establishment, and it retains the biological characteristics of glioma well. Glioma cell transplantation model and transgene model are the most widely used glioma models [50, 51]. Therefore, in this paper, GL261/TR cells were implanted into white matter of mice brain to produce orthotopic TMZ-resistant glioma, then the therapeutic effect of OXA@Fn on the orthotopic TMZ-resistant glioma was investigated.

**Fig. 8:**
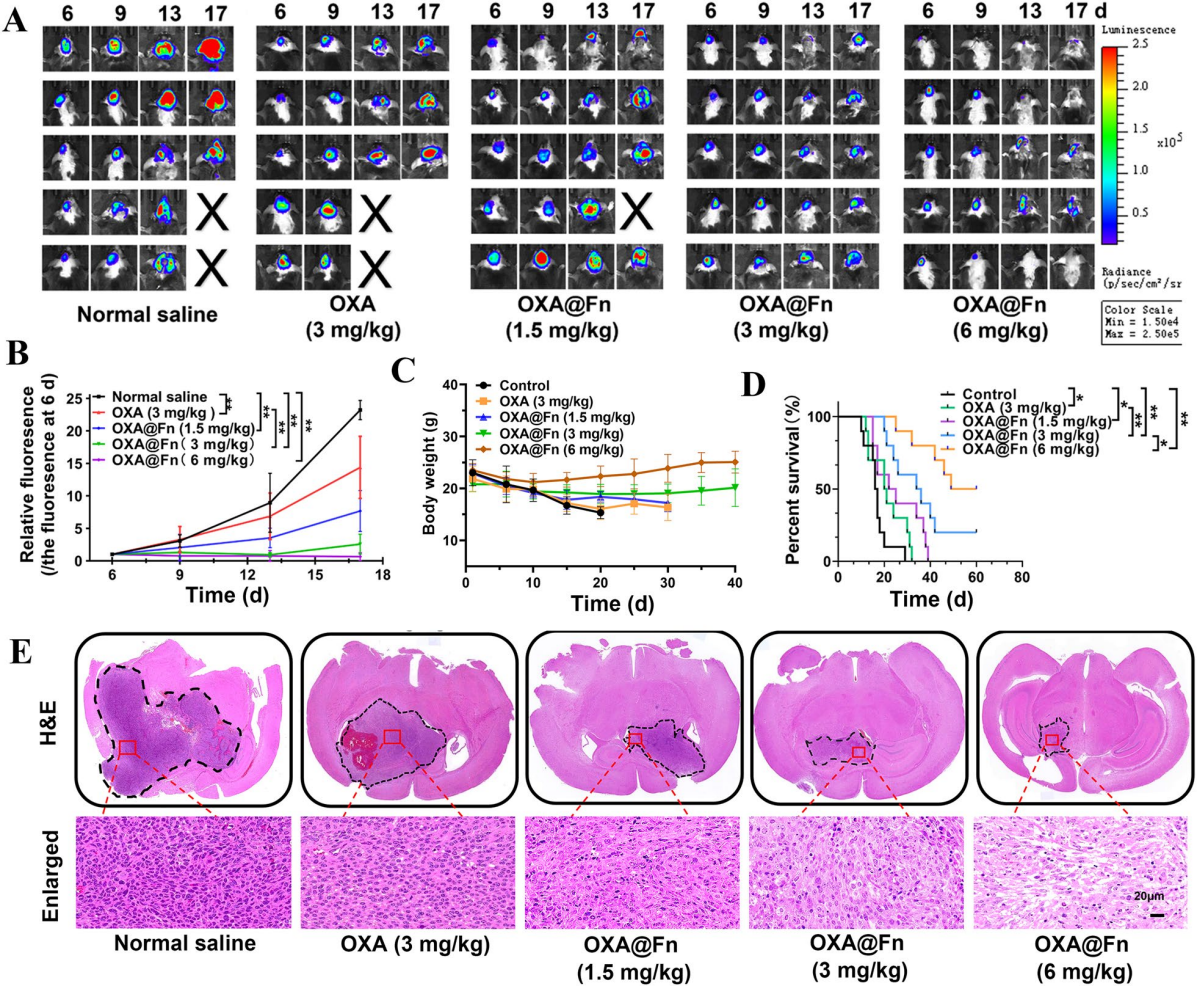
The therapeutic effect of OXA@Fn on orthotopic TMZ-resistant glioma in mice. **A** The inhibitory effect of OXA@Fn on the growth of orthotopic TMZ-resistant glioma observed by in vivo bioluminescence imaging. **B** Statistical analysis of orthotopic TMZ-resistant glioma growth. (n = 5, mean ± SD, *P < 0.05, **P < 0.01). **C** Effects of OXA@Fn on the body weight of orthotopic TMZ-resistant glioma mice. (n = 10, mean ± SD, *P < 0.05, **P < 0.01). **D** Effects of OXA@Fn on the survival time of orthotopic TMZ-resistant glioma mice. (n = 10, mean ± SD, *P < 0.05, **P < 0.01). **E** The H&E stainning of orthotopic TMZ-resistant glioma tissue slices

The inhibitory effect of OXA@Fn on the growth of orthotopic TMZ-resistant glioma was firstly observed by in vivo imager. The results showed that as compared with the normal saline, OXA@Fn inhibited the growth of orthotopic TMZ-resistant glioma in dose-dependent manner, and the inhibitory effect of OXA@Fn was stronger than OXA at the same dose (Fig. 8A, B). After treatment with normal saline and OXA, the body weight of orthotopic TMZ-resistant glioma mice gradually decreased, and OXA@Fn effectively prevented the body weight loss of orthotopic TMZ-resistant glioma mice. Thus, OXA@Fn significantly prolonged the survival time of orthotopic TMZ-resistant glioma mice (Fig. 8C, D). H&E staining also showed that OXA@Fn significantly reduced the volume of orthotopic glioma (Fig. 8E).

**Fig. 9:**
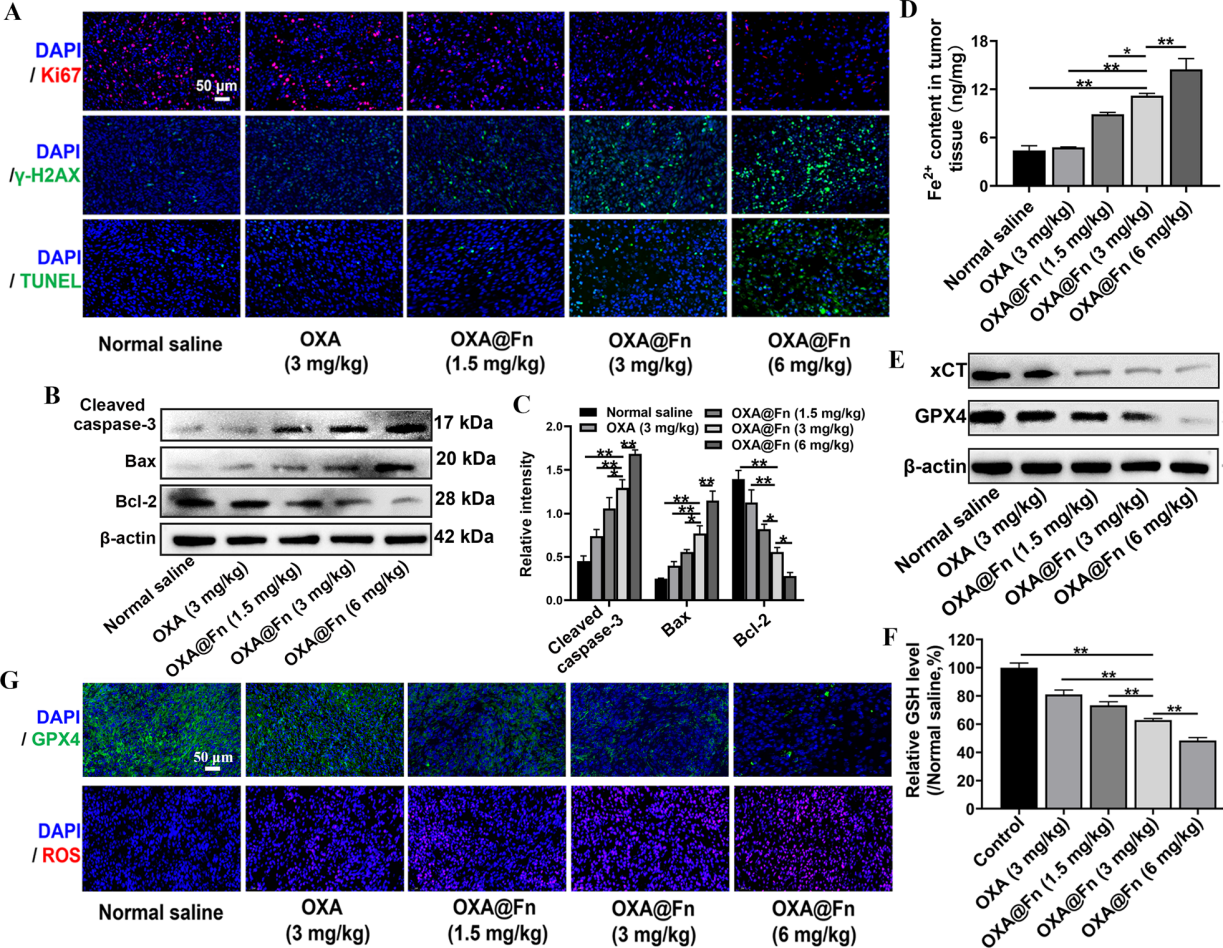
Effect of OXA@Fn on the apoptosis and ferroptosis of orthotopic TMZ-resistant glioma. **A** The images of orthotopic TMZ-resistant glioma tissue slices stained with Ki67 antibody, γ-H2AX antibody and TUNEL assay kit. **B** The expression of apoptosis-related proteins in orthotopic TMZ-resistant glioma tissue. **C** Semi-quantitative analysis of apoptosis-related proteins. **D** The content of Fe^2+^ in orthotopic TMZ-resistant glioma tissue. **E** The expression of xCT and GPX4 in orthotopic TMZ-resistant glioma tissue. **F** The GSH level in orthotopic TMZ-resistant glioma tissue. **G** The images of orthotopic TMZ-resistant glioma tissue slices stained with ROS assay kit and GPX4 antibody. (n = 3, mean ± SD, *P <0.05,**P < 0.01)

The results of immunofluorescence showed that as compared with the normal saline and OXA, OXA@Fn markedly inhibited the expression of Ki67 while increased the level of γ-H2AX and the number of TUNEL positive cells in orthotopic TMZ-resistant glioma tissues (Fig. 9A). Western blot results showed that OXA@Fn decreased the expression of Bcl-2 and increased the expression of cleaved caspase-3 and Bax in orthotopic TMZ-resistant glioma tissues (Fig. 9B, C). These results indicated that OXA@Fn inhibited the growth of orthotopic TMZ-resistant glioma by inducing damage of DNA and apoptosis of orthotopic TMZ-resistant glioma cells. In addition, OXA@Fn significantly increased the expression of E-cadherin and decreased the expression of TGF-β, MMP-9, CD44 and N-cadherin in orthotopic TMZ-resistant glioma tissues (Additional file 1: Fig. S7). These results indicated that OXA@Fn inhibited the invasion of orthotopic TMZ-resistant glioma cells by regulating the expression of invasion-related proteins. Furthermore, OXA@Fn dose-dependently increased the content of Fe^2+^ and ROS and reduced the content of GSH in orthotopic TMZ-resistant glioma tissue, indicating the redox balance in orthotopic TMZ-resistant glioma was badly broken by OXA@Fn. Besides, OXA@Fn also dose-dependently decreased the expression of x-CT and GPX4 in orthotopic TMZ-resistant glioma tissue (Fig. 9D–G, Additional file 1: Fig. S8), which boosted the ferroptosis of orthotopic TMZ-resistant glioma cells. The above data suggested that OXA@Fn caused strong apoptosis and ferroptosis of orthotopic TMZ-resistant glioma tissues. OXA@Fn inhibited the growth of orthotopic TMZ-resistant glioma and prolong the survival time of glioma mice through promoting DNA damage, apoptosis and ferroptosis of orthotopic TMZ-resistant glioma cells.

### Reversion of the immunosuppressive microenvironment in orthotopic TMZ-resistant glioma in mice

**Fig. 10:**
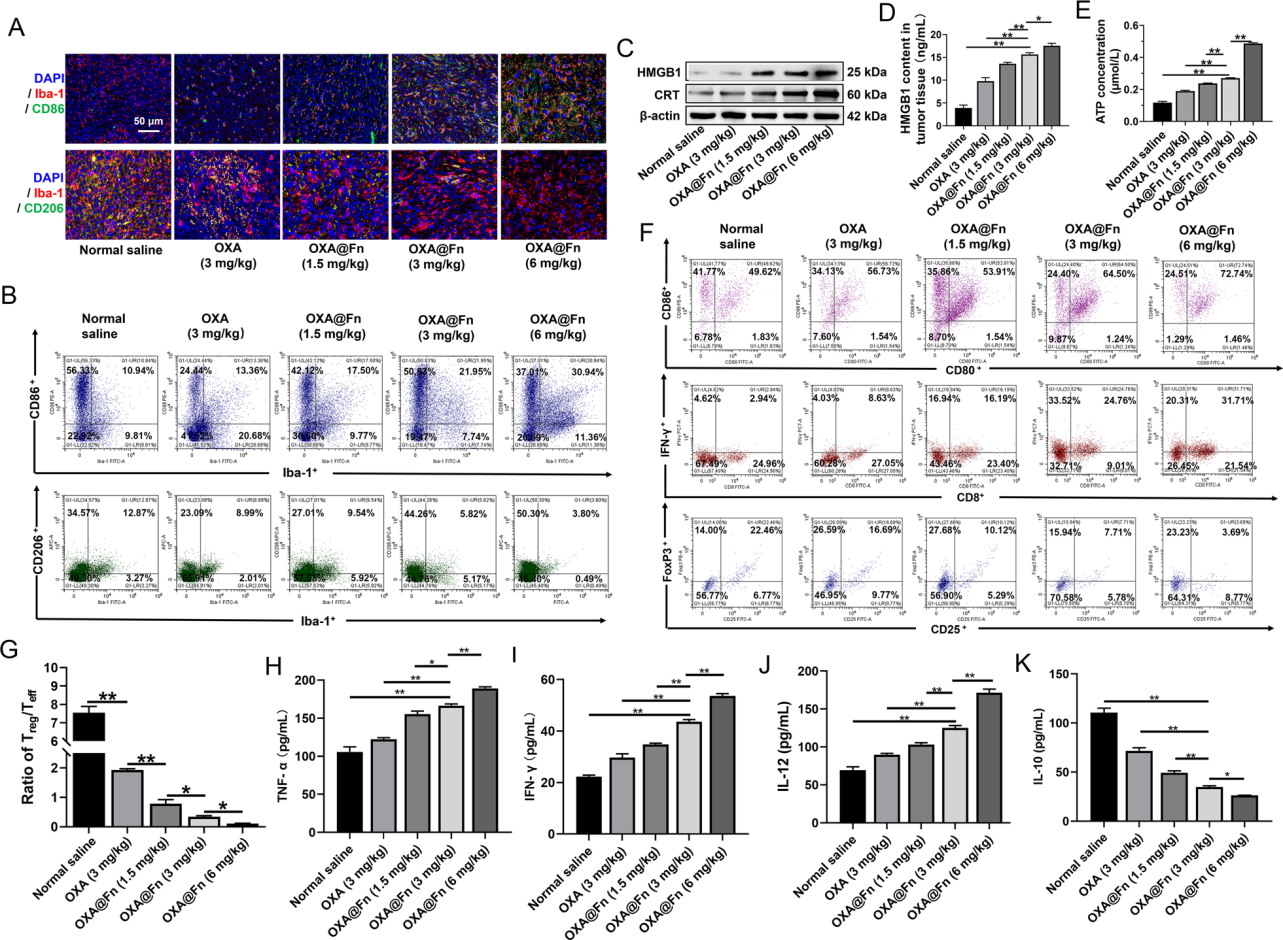
Effect of OXA@Fn on immune microenvironment in orthotopic TMZ-resistant glioma tissue. **A** M1 type GAM (Iba-1 and CD86 co-positive cells) and M2 type GAM (Iba-1 and CD206 co-positive cells) in orthotopic TMZ-resistant glioma tissue. **B** Typical flow cytometric graph of M1 type and M2 type GAM in orthotopic TMZ-resistant glioma tissue. **C** CRT and HMGB1 contents in orthotopic TMZ-resistant glioma tissue detected by western blot. **D**, **E** The HMGB1 and ATP contents in orthotopic TMZ-resistant glioma tissue. **F** Typical flow cytometric graph of the matured DC cells, T_eff_ cells (CD3^+^CD8^+^IFN-γ^+^ T cells) and T_reg_ cells (CD4^+^CD25^+^FoxP3^+^ T cells). **G** The ratio of T_reg_/T_eff_ cells in orthotopic TMZ-resistant glioma tissue. **H**–**K** The content of TNF-α, IFN-γ, IL-12 and IL-10 in orthotopic TMZ-resistant glioma tissues. (n = 3, mean ± SD, *P < 0.05, **P < 0.01)

Immunosuppressive M2 type GAMs account for 30%-50% of the parenchyma of glioma, and it is closely related to the occurrence, development, angiogenesis, invasion and metastasis of glioma [52, 53]. It has been reported that ferroptosis can promote the polarization of macrophages towards M1 phenotype in tumor microenvironment [21, 54]. Therefore, we investigated the effect of OXA@Fn on GAMs polarization in orthotopic TMZ-resistant glioma tissues. As compared with normal saline and OXA, OXA@Fn dose-dependently increased the number of M1-type GAMs (Iba-1^+^CD86^+^ cells) and decreased the number of M2-type GAMs (Iba-1^+^CD206^+^ cells) in orthotopic TMZ-resistant glioma tissue (Fig. 10A, B, Additional file 1: Fig. S9). These results suggested that OXA@Fn induced the polarization of GAMs into M1 type and reversed the immunosuppressive microenvironment by increasing the proportion of M1 type GAMs in orthotopic TMZ-resistant glioma tissue.

After the treatment of OXA@Fn, the expression of CRT and the release of HMGB1 and ATP were increased in orthotopic TMZ-resistant glioma tissue (Fig. 10C–E, Additional file 1: Fig. S10). Flow cytometer results showed that the proportions of CD11c^+^CD80^+^CD86^+^ in orthotopic TMZ-resistant glioma tissue were 48.98%, 57.31%, 54.66%, 64.32% and 73.68% in normal saline, OXA (3 mg/kg), OXA@Fn (1.5 mg/kg), OXA@Fn (3 mg/kg) and OXA@Fn (6 mg/kg) group, respectively (Fig. 10F, Additional file 1: Fig. S11A). These results suggested that OXA@Fn significantly promoted the maturation of DC cells in orthotopic TMZ-resistant glioma tissue by inducing the exposure of CRT and the release of HMGB1 and ATP from orthotopic TMZ-resistant glioma cell. The above data demonstrated that OXA@Fn reversed the immunosuppressive microenvironment by promoting the maturation of DC cells in orthotopic TMZ-resistant glioma tissue.

Studies have shown that matured DC cells and M1 type GAMs are able to present antigens to T cells, thus promoting the activation of T cell [23, 55]. Flow cytometer results showed that in normal saline, OXA (3 mg/kg), OXA@Fn (1.5 mg/kg), OXA@Fn (3 mg/kg) and OXA@Fn (6 mg/kg) treatment group, the proportion of CD3^+^CD8^+^IFN-γ^+^ T cells (T_eff_ cells) in orthotopic TMZ-resistant glioma tissue was 3.00%, 8.22%, 15.57%, 25.03% and 32.73%, respectively. The proportion of CD4^+^CD25^+^FoxP^3+^ T cells (T_reg_ cells) in orthotopic TMZ-resistant glioma tissue was 23.93%, 15.83%, 12.14%, 8.54% and 3.60%, respectively (Fig. 10F, G, Additional file 1: Fig. S11B, C). These results indicated that OXA@Fn increased the proportion of T_eff_ cells and reduced the proportion of T_reg_ cells in orthotopic TMZ-resistant glioma tissue in dose-dependent manner, indicating OXA@Fn reversed the immunosuppressive microenvironment by increasing the proportion T_eff_ cells in orthotopic TMZ-resistant glioma tissue. At the same time, OXA@Fn dose-dependently increased the contents of TNF-α, IFN-γ, IL-12 and decreased the content of IL-10 in orthotopic TMZ-resistant glioma tissue (Fig. 10H–K), suggesting the immunosuppressive microenvironment was reversed by OXA@Fn. In a word, these results demonstrated that OXA@Fn augmented ferroptosis of TMZ-resistant glioma cells, subsequently promoted the maturation of DC cells. Then matured DC cells and M1-type GAMs presented antigen to T cells and subsequently activated cytotoxic T lymphocytes. Consequently, the immunosuppressive microenvironment in orthotopic TMZ-resistant glioma tissue was reversed and the growth and invasion of orthotopic TMZ-resistant glioma was synergistically inhibited.

In our previous research [4], siRNA was used to silence the expression of PD-L1 and block the PD-1/PD-L1 signaling pathway in orthotopic TMZ-resistant glioma, thereby increasing the infiltration and activity of T_eff_ cells and reversing the immunosuppressive microenvironment in orthotopic TMZ-resistant glioma tissue. In this paper, although OXA@Fn did not target the PD-1/PD-L1 signaling pathway, it promoted ferroptosis of orthotopic TMZ-resistant glioma tissue, which subsequently induced the maturation of DC cells and activated cytotoxic T lymphocytes to inhibit the growth of orthotopic TMZ-resistant glioma. In addition, in our previous research, ROS in orthotopic TMZ-resistant glioma was depleted by drug carrier material, which subsequently decreased O^6^-methyl-guanine-DNA methyltransferase (MGMT) expression and enhanced the therapeutic effect of TMZ on orthotopic TMZ-resistant glioma. The mechanism of action of OXA@Fn on orthotopic TMZ-resistant glioma is different from that of TMZ, so OXA@Fn could inhibit the growth of orthotopic TMZ-resistant glioma.

### Preliminary safety evaluation of OXA@Fn

**Fig. 11:**
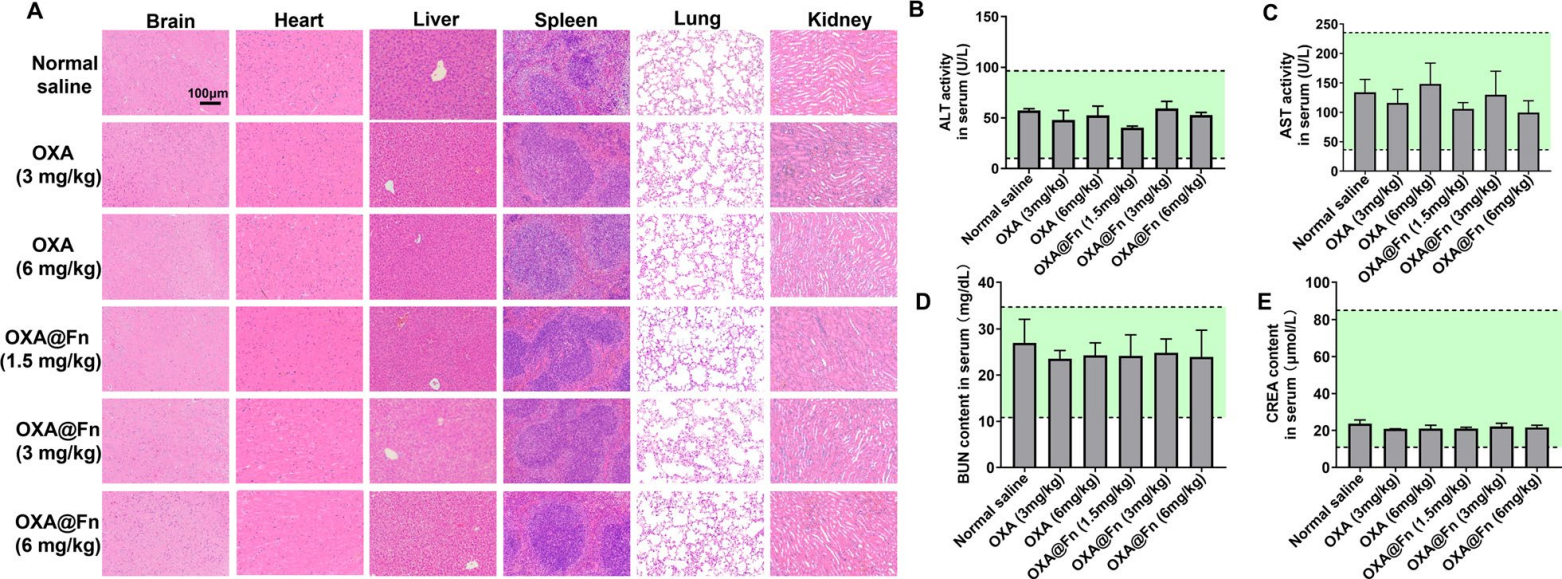
Preliminary safety evaluation of OXA@Fn in mice. **A** H&E staining of brain, heart, liver, spleen, lung and kidney tissue in mice. **B**, **C** Effects of OXA@Fn on ALT and AST activity in serum of mice. **D**, **E** Effects of OXA@Fn on content of BUN and CREA in serum of mice. The green area indicates the normal ranges. (n = 3, mean ± SD)

H&E staining showed that no significant morphological abnormalities were observed in brain, heart, liver, spleen, lung and kidney in OXA@Fn treated mice (Fig. 11A). Biochemical analysis further showed that ALT and AST activities, BUN and CREA contents in mice serum were all within the normal range (Fig. 11B–E), indicating that OXA@Fn did not cause obvious systemic toxicity in mice at the therapeutic dose.

## Conclusions

In OXA@Fn, Fn was a delivery vector of OXA, which helped OXA cross BBB and target to glioma cells. Fn could also induce ferroptosis of TMZ-resistant glioma cells. OXA not only induced apoptosis of TMZ-resistant glioma cells but also promoted the production of H_2_O_2_. OXA@Fn specifically delivered Fn and OXA to orthotopic TMZ-resistant glioma tissue in mice via TfR1, and it promoted the apoptosis and boosted ferroptosis of TMZ-resistant glioma cells by locking up Fe^2+^ inside the TMZ-resistant glioma cells. Besides, OXA@Fn significantly reversed the immunosuppressant microenvironment in orthotopic TMZ-resistant glioma by promoting the maturation of DC cells, inducing the polarization of GAMs into M1 type and activating toxic T lymphocytes in orthotopic glioma tissues. In a word, by promoting the apoptosis and boosting ferroptosis of glioma cells synergistically, and also by reversing the immunosuppressive microenvironment in orthotopic TMZ-resistant glioma tissues, OXA@Fn inhibited the growth and invasion of orthotopic TMZ-resistant glioma. OXA@Fn has the advantages of simple structure, easy preparation and good safety, and it shows great potential in clinical transformation.

### Supplementary Information

**Additional file 1: Figure S1.** X-ray photoelectron spectroscopy (XPS) image of OXA@Fn. (A) XPS image of carbon element in OXA@Fn. (B) XPS image of oxygen element in OXA@Fn. (C) XPS image of nitrogen element in OXA@Fn. (D) XPS image of iron element in OXA@Fn. (E) XPS image of platinum element in OXA@Fn. **Figure S2.** Uptake of OXA@Fn by GL261/TR cells in the presence of different uptake inhibitors. (A) Uptake of OXA@Fn by GL261/TR cells in the presence of different uptake inhibitors observed by CLSM. (B) The typical flow cytometer diagrams of the uptake of OXA@Fn by GL261/TR cells in the presence of different uptake inhibitors. (C) Statistical analysis of the effect of different uptake inhibitors and transferrin on uptake of Cy5 labeled OXA@Fn by GL261/TR cells detected by flow cytometer. (n = 3, mean ± SD, **P < 0.01). **Figure S3.** The effect of OXA@Fn on the viability of GL261/TR cells. (A) The death ratio of GL261/TR cells. (B) The cloning formation rate of GL261/TR cells after cells were treated with OXA@Fn. (C) The typical flow cytometer diagram of LPO in GL261/TR cells. (D) Statistical analysis of the LPO in GL261/TR cells. (E) The typical flow cytometer diagram of ROS in GL261/TR cells. (F) Statistical analysis of the ROS in GL261/TR cells. (n = 3, mean ± SD, *P < 0.05, **P < 0.01). **Figure S4.** The resistance values between transwell donor chamber and recipient chamber before and after drug administration. (n = 3, mean ± SD, ns: no significant difference). **Figure S5.** The expression of TfR1 in bEnd 3 cells and HUVEC. **Figure S6.** The integrated brain targeting efficiency of OXA@Fn. **Figure S7.** The expression of invasion-related proteins in orthotopic TMZ-resistant glioma tissue after the treatment of OXA@Fn. (A) The expression of invasion-related proteins in orthotopic TMZ-resistant glioma tissue detected by western blot. (B) Semi-quantitative analysis of invasion-related proteins. (n = 3, mean ± SD, *P < 0.05, **P < 0.01). **Figure S8.** Semi-quantitative analysis of xCT and GPX4 expression in orthotopic TMZ-resistant glioma tissue. (n = 3, mean ± SD, *P < 0.05, **P < 0.01). **Figure S9.** Effect of OXA@Fn on polarization of glioma-associated macrophages (GAM) in orthotopic TMZ-resistant glioma tissue. (A) Statistic analysis of M1 type GAM (Iba-1 and CD86 co-positive cells) in orthotopic TMZ-resistant glioma tissue. (B) Statistic analysis of M2 type GAM (Iba-1 and CD206 co-positive cells) in orthotopic TMZ-resistant glioma tissue. (n = 3, mean ± SD, *P < 0.05, **P < 0.01). **Figure S10.** Semi-quantitative analysis of CRT and HMGB1 expression in orthotopic TMZ-resistant glioma tissue. (n = 3, mean ± SD, *P < 0.05, **P < 0.01). **Figure S11.** Effect of OXA@Fn on immune microenvironment in orthotopic TMZ-resistant glioma tissue. (A) The ratio of matured DC cell in orthotopic TMZ-resistant glioma tissue. (B) The proportion of T_eff_ cell in orthotopic TMZ-resistant glioma tissue. (C) The proportion of T_reg_ cell in orthotopic TMZ-resistant glioma tissue. (n = 3, mean ± SD, *P < 0.05, **P < 0.01).

## Data Availability

All data generated or analyzed during this study are included in this published article and its Additional file.

## Abbreviations

ATP: Adenosine-triphosphate
ALT: Alanine aminotransferase
AST: Aspartate aminotransferase
BBB: Blood brain barrier
BODIPY-C11: Boron difluoride pyrrole fluorescent dyes-C11
BUN: Urea nitrogen
BSA: Bovine albumin
CD11c-APC: Blusters of differentiation 11 c-Allophycocyanin
CD25-FITC: Blusters of differentiation 25-fluorescein isothiocyanate
CD3-APC: Blusters of differentiation 3-allophycocyanin
CD31: Platelet endothelial cell adhesion molecule-1
CD4-PE-Cy7: Clusters of differentiation4-phycoerythrin-sulfo-cyanine7
CD44: Clusters of differentiation 44
CD8-FITC: Clusters of differentiation 8-fluorescein isothiocyanate
CD80-FITC: Clusters of differentiation 80-fluorescein
CD86-PE: Clusters of differentiation86-phycoerythrin
CREA: Creatinine
CRT: Calreticulin
DAPI: 4′,6-Diamidino-2-phenylindole dihydrochloride
DCFH-DA: 2,7-Dichlorodihydrofluorescein diacetate
DFO: Deferoxamine
DHE: Dihydroethidium
DMEM: Dulbecco’s modified eagle medium
DMSO: Dimethyl sulfoxide
E-cadherin: E-Ca^2+^ dependent cell adhesion molecules
FoxP3-PE: Forkhead box P3-phycoerythrin
Fer-1: Ferrostatin-1
Fn: Ferritin
FPN1: Ferroportin 1
GL261/TR: TMZ-resistant glioma cells
GSH: Glutathione
GM-CSF: Granulocyte–macrophage colony stimulating factor
GPX4: Glutathione peroxidase 4
H&E staining: Hematoxylin–eosin staining
HMGB1: Human high-mobility group box-1
Iba-1: Ionized calcium binding adaptor molecule 1
Iba-1: Ionized calcium binding adaptor molecule 1
IL-6/10/12: Interleukin-6/10/12
IFN-γ: Interferon-γ
IFN-γ-PE-Cy7: Interferon-γ-phycoerythrin-sulfo-cyanine7
LPO: Lipid peroxidation
LSCM: Laser scanning confocal microscopy
MTT: 3-(4,5-Dimethylthiazol-2-yl)-2,5-diphenyltetrazolium bromide
N-cadherin: N-Ca^2+^ dependent cell adhesion molecules
NK cells: Natural killer cell
OXA: Oxaliplatin
PBS: Phosphate buffer saline
PDI: Polydispersity index
PD-L1: Programmed cell death ligand-1
ROS: Reactive oxygen species
RPMI1640: Roswell Park Memorial Institute 1640
SDS-PAGE: Sulfate-polyacrylamide gel electrophoresis
TEM: Transmission electron microscope
TfR1: Transferrin receptor 1
TGF-β: Transforming growth factor-β
TNF-α: Tumor necrosis factor-α
T_reg_: Regulatory T cell
Tris: Trimethylol aminomethane
xCT: Cystine/glutamic acid reverse transporter
