# Supplementary material for: Glioma-targeted oxaliplatin/ferritin clathrate reversing the immunosuppressive microenvironment through hijacking Fe2+ and boosting Fenton reaction
Source: J Nanobiotechnology. 2024 Mar 5;22:93. doi: 10.1186/s12951-024-02376-w (PMC10913265; doi:10.1186/s12951-024-02376-w)
Supplement: Supplementary file 1 — Additional file 1: Figure S1. X-ray photoelectron spectroscopy (XPS) image of OXA@Fn. (A) XPS image of carbon element in OXA@Fn. (B) XPS image of oxygen element in OXA@Fn. (C) XPS image of nitrogen element in OXA@Fn. (D) XPS image of iron element in OXA@Fn. (E) XPS image of platinum element in OXA@Fn. Figure S2. Uptake of OXA@Fn by GL261/TR cells in the presence of different uptake inhibitors. (A) Uptake of OXA@Fn by GL261/TR cells in the presence of different uptake inhibitors observed by CLSM. (B) The typical flow cytometer diagrams of the uptake of OXA@Fn by GL261/TR cells in the presence of different uptake inhibitors. (C) Statistical analysis of the effect of different uptake inhibitors and transferrin on uptake of Cy5 labeled OXA@Fn by GL261/TR cells detected by flow cytometer. (n = 3, mean ± SD, **P < 0.01). Figure S3. The effect of OXA@Fn on the viability of GL261/TR cells. (A) The death ratio of GL261/TR cells. (B) The cloning formation rate of GL261/TR cells after cells were treated with OXA@Fn. (C) The typical flow cytometer diagram of LPO in GL261/TR cells. (D) Statistical analysis of the LPO in GL261/TR cells. (E) The typical flow cytometer diagram of ROS in GL261/TR cells. (F) Statistical analysis of the ROS in GL261/TR cells. (n = 3, mean ± SD, *P < 0.05, **P < 0.01). Figure S4. The resistance values between transwell donor chamber and recipient chamber before and after drug administration. (n = 3, mean ± SD, ns: no significant difference). Figure S5. The expression of TfR1 in bEnd 3 cells and HUVEC. Figure S6. The integrated brain targeting efficiency of OXA@Fn. Figure S7. The expression of invasion-related proteins in orthotopic TMZ-resistant glioma tissue after the treatment of OXA@Fn. (A) The expression of invasion-related proteins in orthotopic TMZ-resistant glioma tissue detected by western blot. (B) Semi-quantitative analysis of invasion-related proteins. (n = 3, mean ± SD, *P < 0.05, **P < 0.01). Figure S8. Semi-quantitative analysis of xCT an [file 12951_2024_2376_MOESM1_ESM.docx]

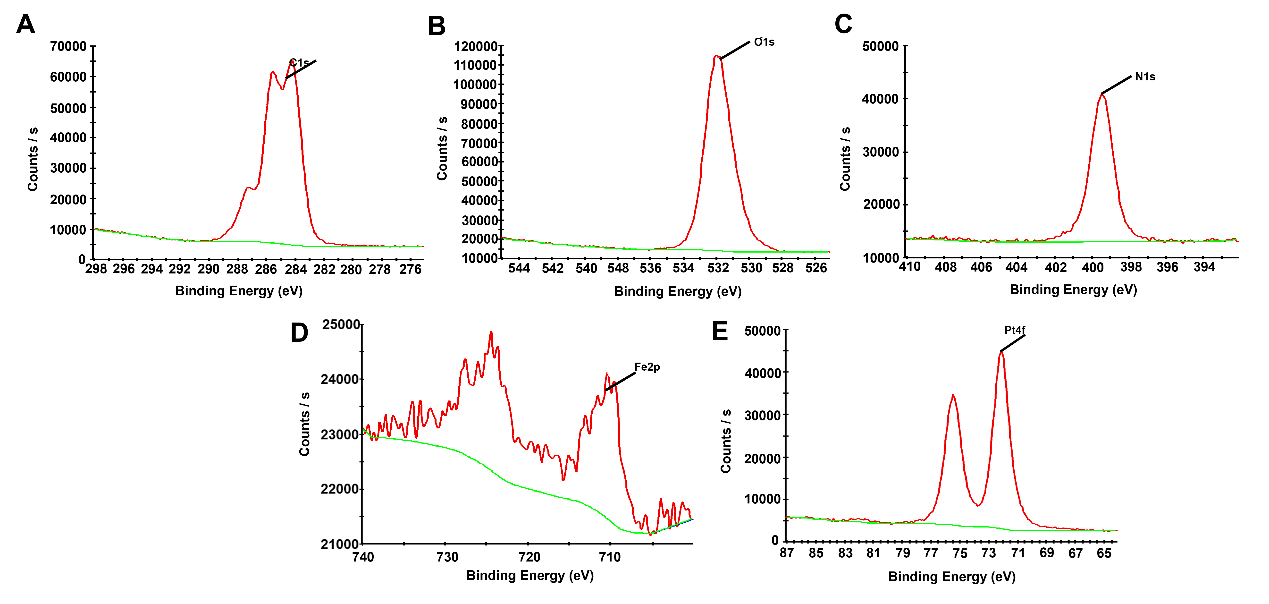


**Figure S1.** X-ray photoelectron spectroscopy (XPS) image of OXA@Fn. (A) XPS image of carbon element in OXA@Fn. (B) XPS image of oxygen element in OXA@Fn. (C) XPS image of nitrogen element in OXA@Fn. (D) XPS image of iron element in OXA@Fn. (E) XPS image of platinum element in OXA@Fn.

**
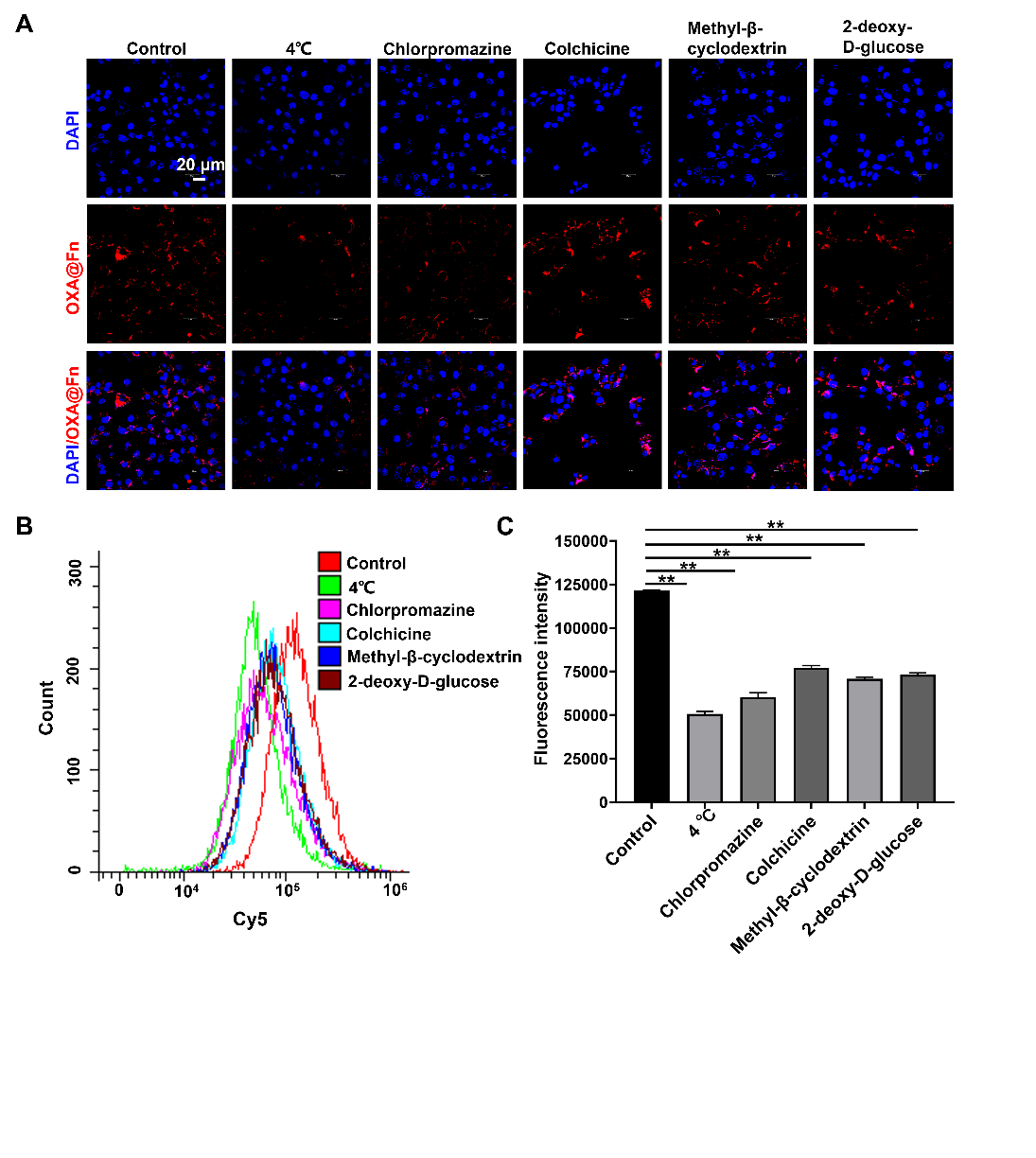
**

**Figure S2.** Uptake of OXA@Fn by GL261/TR cells in the presence of different uptake inhibitors. (**A)** Uptake of OXA@Fn by GL261/TR cells in the presence of different uptake inhibitors observed by CLSM. (**B)** The typical flow cytometer diagrams of the uptake of OXA@Fn by GL261/TR cells in the presence of different uptake inhibitors. (**C)** Statistical analysis of the effect of different uptake inhibitors and transferrin on uptake of Cy5 labeled OXA@Fn by GL261/TR cells detected by flow cytometer. (n=3, mean ± SD, ***P*<0.01).


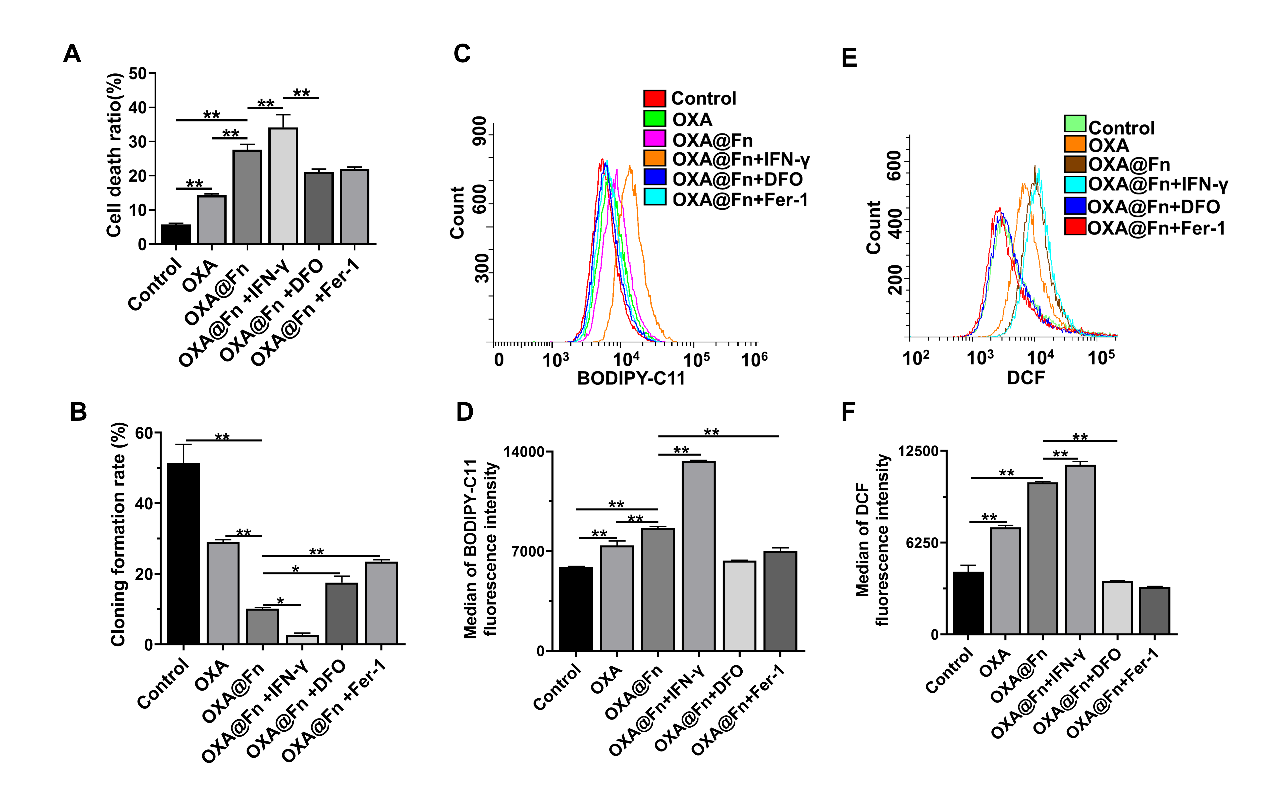


**Figure S3.** The effect of OXA@Fn on the viability of GL261/TR cells. (A) The death ratio of GL261/TR cells. (B) The cloning formation rate of GL261/TR cells after cells were treated with OXA@Fn. (C) The typical flow cytometer diagram of LPO in GL261/TR cells. (D) Statistical analysis of the LPO in GL261/TR cells. (E) The typical flow cytometer diagram of ROS in GL261/TR cells. (F) Statistical analysis of the ROS in GL261/TR cells. (n=3, mean ± SD, ^*^*P*<0.05, ^**^*P*<0.01).


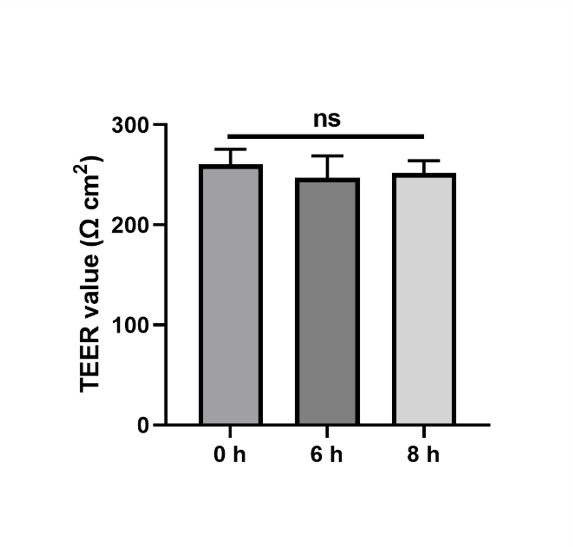


**Figure S4.** The resistance values between transwell donor chamber and recipient chamber before and after drug administration. (n=3, mean ± SD, ns: no significant difference).


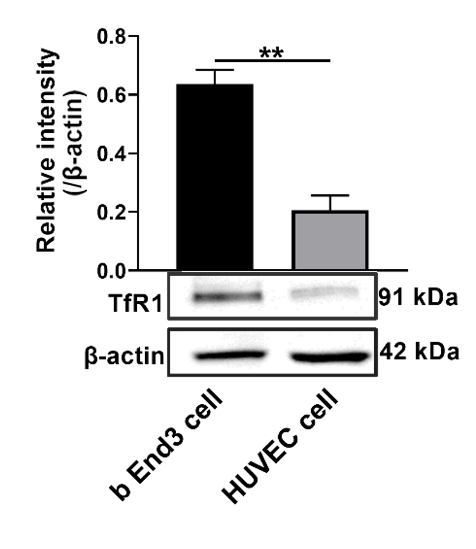


**Figure S5.** The expression of TfR1 in bEnd 3 cells and HUVEC.


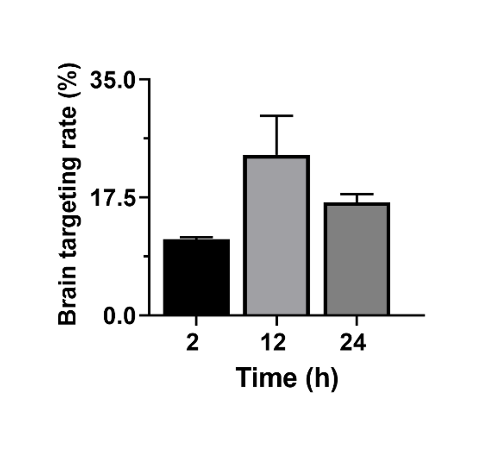


**Figure S6.** The integrated brain targeting efficiency of OXA@Fn.


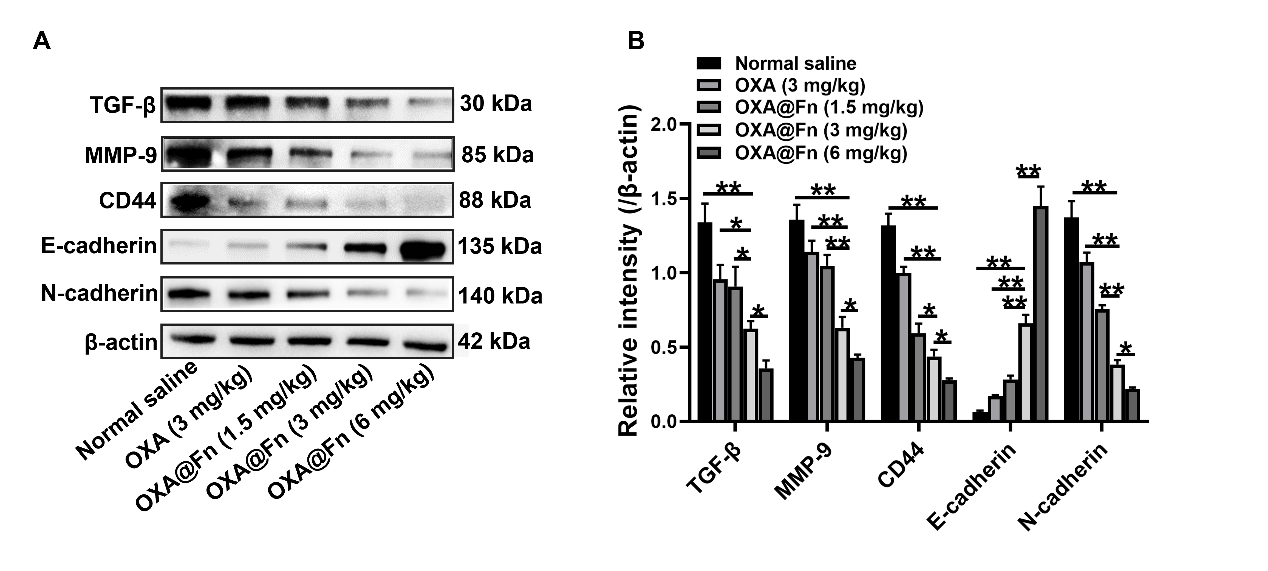


**Figure S7.** The expression of invasion-related proteins in orthotopic TMZ-resistant glioma tissue after the treatment of OXA@Fn. (A) The expression of invasion-related proteins in orthotopic TMZ-resistant glioma tissue detected by western blot. (B) Semi-quantitative analysis of invasion-related proteins. (n=3, mean ± SD, ^*^*P*<0.05, ^**^*P*<0.01).


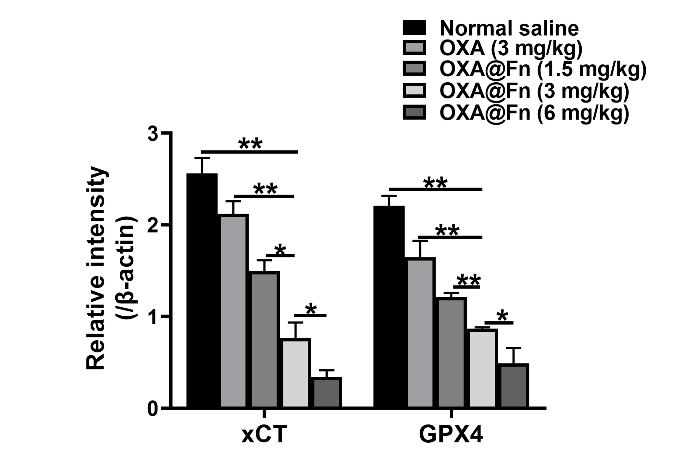


**Figure S8.** Semi-quantitative analysis of xCT and GPX4 expression in orthotopic TMZ-resistant glioma tissue. (n=3, mean±SD, ^*^*P*<0.05, ^**^*P*<0.01).


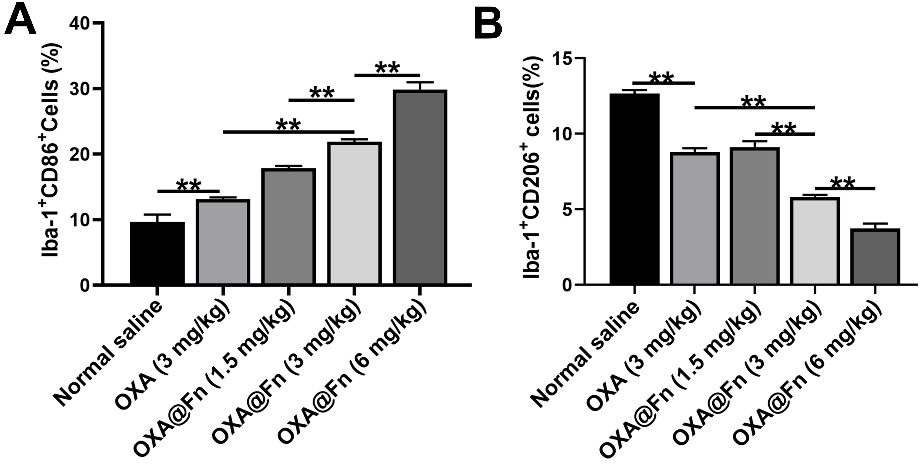


**Figure S9.** Effect of OXA@Fn on polarization of glioma-associated macrophages (GAM) in orthotopic TMZ-resistant glioma tissue. (A) Statistic analysis of M1 type GAM (Iba-1 and CD86 co-positive cells) in orthotopic TMZ-resistant glioma tissue. (B) Statistic analysis of M2 type GAM (Iba-1 and CD206 co-positive cells) in orthotopic TMZ-resistant glioma tissue. (n=3, mean±SD, ^*^*P*<0.05, ^**^*P*<0.01).


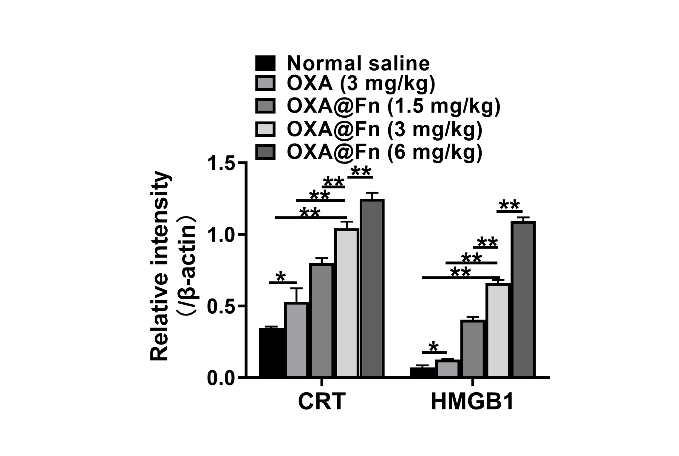


**Figure S10.** Semi-quantitative analysis of CRT and HMGB1 expression in orthotopic TMZ-resistant glioma tissue. (n=3, mean±SD, **P*<0.05, ***P*<0.01).

**
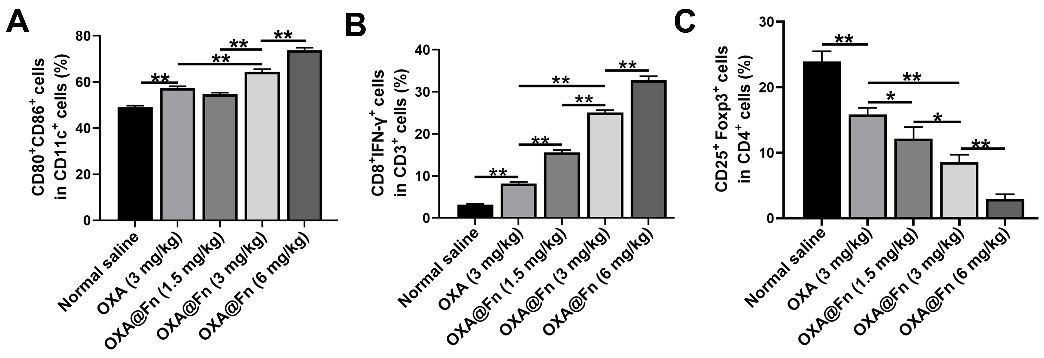
**

**Figure S11.** Effect of OXA@Fn on immune microenvironment in orthotopic TMZ-resistant glioma tissue. **(A)** The ratio of matured DC cell in orthotopic TMZ-resistant glioma tissue. **(B)** The proportion of T_eff_ cell in orthotopic TMZ-resistant glioma tissue. **(C)** The proportion of T_reg_ cell in orthotopic TMZ-resistant glioma tissue. (n=3, mean ± SD, ^*^*P*<0.05, ^**^*P*<0.01).
